# Feasibility of identifying the ideal locations for motor intention decoding using unimodal and multimodal classification at 7T-fMRI

**DOI:** 10.1038/s41598-018-33839-4

**Published:** 2018-10-22

**Authors:** Peter E. Yoo, Thomas J. Oxley, Sam E. John, Nicholas L. Opie, Roger J. Ordidge, Terence J. O’Brien, Maureen A. Hagan, Yan T. Wong, Bradford A. Moffat

**Affiliations:** 1 Department of Anatomy and Neuroscience, The University of Melbourne, VIC, Australia; 2 Vascular Bionics Laboratory, Melbourne Brain Centre, Department of Medicine, Royal Melbourne Hospital, The University of Melbourne, VIC, Australia; 3 The Florey Institute of Neuroscience and Mental Health, VIC, Australia; 4 The Departments of Neuroscience, The Central Clinical School, Monash University, VIC, Australia; 5 The Department of Neurology, the Alfred Hospital, Melbourne, VIC, Australia; 6 Department of Physiology, Monash University, VIC, Australia; 7 Biomedicine Discovery Institute, Monash University, VIC, Australia; 8 Department of Electrical and Computer Systems Engineering, Monash University, VIC, Australia

## Abstract

Invasive Brain-Computer Interfaces (BCIs) require surgeries with high health-risks. The risk-to-benefit ratio of the procedure could potentially be improved by pre-surgically identifying the ideal locations for mental strategy classification. We recorded high-spatiotemporal resolution blood-oxygenation-level-dependent (BOLD) signals using functional MRI at 7 Tesla in eleven healthy participants during two motor imagery tasks. *BCI diagnostic task* isolated the intent to imagine movements, while *BCI simulation task* simulated the neural states that may be yielded in a real-life BCI-operation scenario. Imagination of movements were classified from the BOLD signals in sub-regions of activation within a single or multiple dorsal motor network regions. Then, the participant’s decoding performance during the BCI simulation task was predicted from the BCI diagnostic task. The results revealed that drawing information from multiple regions compared to a single region increased the classification accuracy of imagined movements. Importantly, systematic unimodal and multimodal classification revealed the ideal combination of regions that yielded the best classification accuracy at the individual-level. Lastly, a given participant’s decoding performance achieved during the BCI simulation task could be predicted from the BCI diagnostic task. These results show the feasibility of 7T-fMRI with unimodal and multimodal classification being utilized for identifying ideal sites for mental strategy classification.

## Introduction

Invasive Brain-Computer Interfaces (BCIs) can capture, characterize and translate neural states (i.e., classify) underlying a mental strategy to grant paralysed individuals direct brain-control of assistive devices. However, invasive BCIs require implantation of intracranial electrode arrays through open-brain surgery that inevitably exposes significant health risks to the patients^1–7^.

The risk-to-benefit ratio of the procedure could potentially be increased by pre-surgically identifying the ideal cortical location(s) for classifying mental strategies at the individual-level. Currently, a similar pre-surgical procedure is often performed using functional magnetic resonance imaging (fMRI) in weak to moderate field-strength MRI scanners (e.g., 1.5T or 3T; clinical fMRI). Regions of significant blood-oxygenation-level-dependent (BOLD) signal fluctuations (i.e., BOLD activations) are identified to estimate the neural substrates underlying the mental strategy, which can then be used to guide the electrode array implantation^1^.

However, the spatial specificity of the BOLD activation to the underlying neural activity (i.e., BOLD specificity) is known to be poor in clinical fMRI, due to a significant proportion of the signals arising from distant draining veins^8,9^. The poor BOLD specificity decreases the confidence in which electrode implantation sites can be determined based on fMRI. Furthermore, BOLD activation maps alone provide little information about which specific sub-regions process the most characteristic information for classifying the mental strategy. Hence, clinical fMRI is currently used to provide support for implanting the electrodes in the region of interest (ROI) defined *a priori* (e.g., seeing whether the area expected to be the hand-knob of primary motor cortex [M1] activate during imagining of hand movements), instead of investigating the ideal regions for information classification.

fMRI at 7 Tesla (7T-fMRI) offers improved BOLD specificity, as the contribution of signal arising near the large draining veins substantially diminishes^10,11^ and that arising near the microvasculature embedded in the parenchyma increases supralinearly with the magnetic field strength^12^. Previous studies have shown that the spatial patterns of BOLD activation at 7T are highly localized to the underlying population neural activity^13–15^. If indeed the sites of 7T-fMRI BOLD activation are localized to the neural substrates underlying a mental strategy, classifying the corresponding information could reveal ideal regions for information classification. To achieve this, information being processed across multiple cortical regions should to be considered.

While most examples of human BCIs have utilized information from a single motor network structure to classify the mental strategies (i.e. *unimodal classification*)^1–7^, harnessing different types of information processed across many cortical regions (i.e., *multimodal classification*) may improve the robustness of classification also. Consistently, previous non-human primate BCI studies have demonstrated that a variety of motor parameters and an entire arm-reach trajectory sequence in multiple directions could be robustly decoded from signals recorded across multiple motor regions simultaneously^16–18^. However, it is unknown whether BOLD signals are sensitive to the different information processed across different regions, where such information can be decoded.

This study aimed to demonstrate the feasibility of utilizing high spatiotemporal resolution 7T-fMRI with unimodal and multimodal classification to identify the ideal combination of areas for mental strategy classification. In healthy volunteers, significant regions of activation across the dorsal motor network were identified while the participants imagined performing specific movements, using a cued GO/NO-GO task (i.e., *BCI diagnostic task*). Then, each imagined movement was classified against one another based on the corresponding BOLD states yielded at the individual-level. Classification was performed using both unimodal and multimodal approach to determine the region or combination of regions that yielded information that resulted in the most accurate classification results (e.g., decoding performance). The participants also performed a task that attempted to simulate the cognitive states yielded during BCI-control, where they imagined different types of movement consecutively (i.e., *BCI simulation task*). A given individual’s decoding performance yielded during the BCI simulation task was predicted from that yielded during the BCI diagnostic task.

## Results

It is integral to become familiar with the terminology used in this study to comprehend the results effectively. We recommend reading the section Terminology, under Materials and Methods first.

### Regions of BOLD activation for imagined movements can be detected at the individual-level

**Figure 1:**
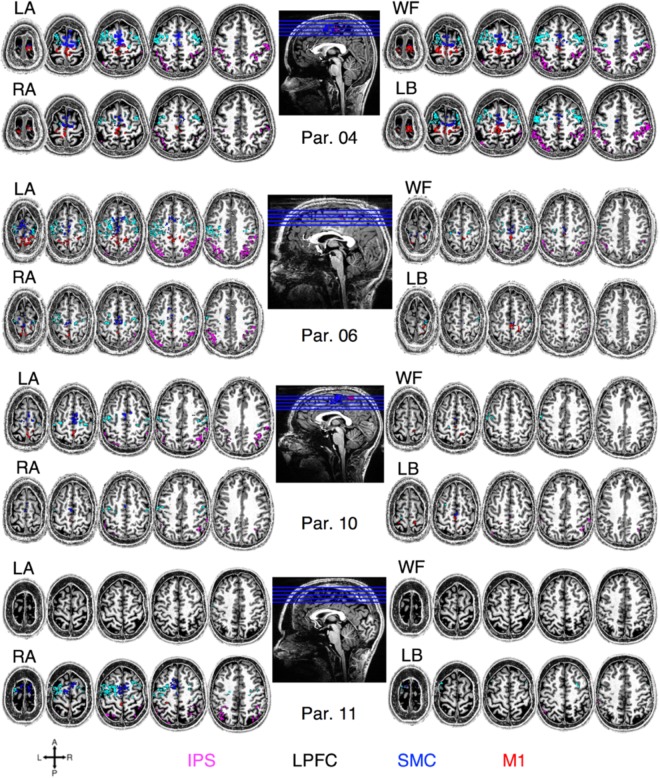
Participant-specific regions of significant BOLD activation during specific imagined movements for representative participants 4, 6, 10 and 11. The figures show regions of significant BOLD activation during the BCI diagnostic task representative participant 4, 6, 10 and 11 for each condition, LA, RA, WF, and LB, in arbitrarily selected slices in the participant’s T_1_-weighted anatomical image space. Significance was determined at *Z* > 2.3, *p* < 0.05, cluster-wise correction. Significant regions of activation within the IPS, the LPFC, the SMC and the M1 are colored in magenta, cyan, blue and red, respectively.

The BCI diagnostic task revealed individual-level differences in the ability to generate significant BOLD activity during imagined movements (Fig. 1). Significant BOLD activation was observed for each of the imagined movement conditions, left ankle (LA), right ankle (RA), walk forward (WF) and lean back (LB), in most participants across spatially distinct sub-regions of the: intraparietal sulcus (IPS; 9/11 participants; condition-mean volume of significant voxels ± standard error averaged across participants; 1313 ± 221 mm^3^); the lateral prefrontal cortex (LPFC; 9/11 participants; 1225 ± 190 mm^3^); the supplementary motor cortex (SMC; 9/11 participants; 776 ± 123 mm^3^); and in the dorsomedial M1 (8/11 participants; 351 ± 76 mm^3^; all *Z* > 2.3, *p* < 0.05 cluster-wise correction). However, the ability to generate robust BOLD activity varied across individuals. For example, the average volume of significant activation across the conditions for participants 4, 6, 10 and 11 were: 2765 mm^3^, 2703 mm^3^, 712 mm^3^ and 991 mm^3^ in the IPS, respectively; 3032 mm^3^, 1994 mm^3^, 623 mm^3^ and 808 mm^3^ in the LPFC, respectively; 2173 mm^3^, 1072 mm^3^, 571 mm^3^, and 537 mm^3^ in the SMC, respectively; and 3032 mm^3^, 1994 mm^3^, 623 mm^3^ and 808 mm^3^ in the M1, respectively (Fig. 1).

### Imagining of specific movements can be decoded from the BOLD signal

**Figure 2:**
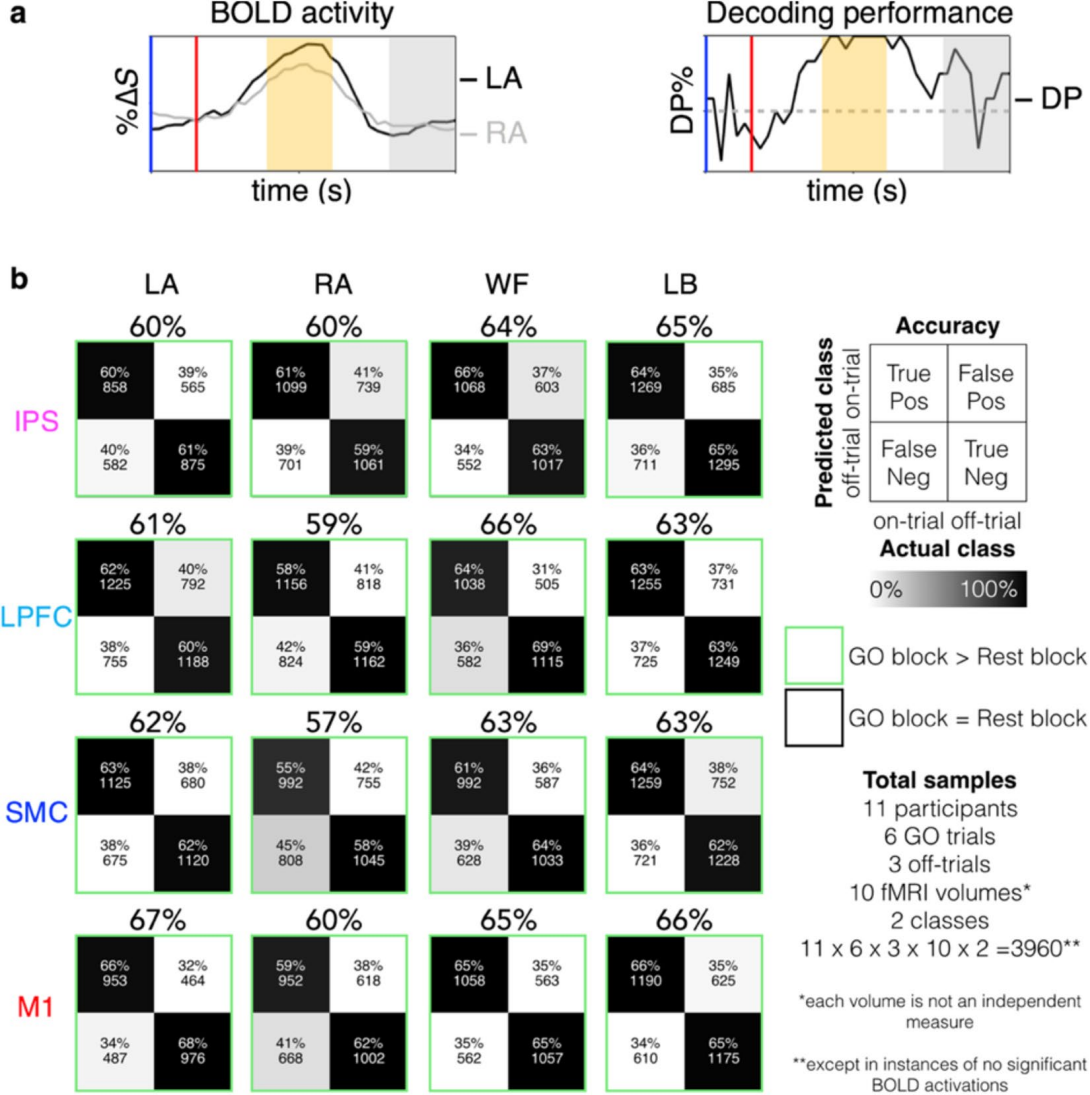
Group-level unimodal classification decoding performance during the BCI diagnostic task. Specific imagined movement conditions, left ankle, right ankle, walk forward, and lean back, could be decoded against each other across the dorsal motor network regions at the group-level. (**a**) A representation of the relationship between the BOLD activity and the decoding performance across time. Decoding performance is expected to increase with the BOLD activity, as there should be greater difference in BOLD %∆*S* between the on and off-trial during imagination compared to rest. An example of when left ankle trial is decoded against the right ankle trial. The upright blue and red bars denote the start of prompt and GO blocks, respectively. The orange and grey shadings denote the time-points that correspond to the GO block and REST block, respectively. (**b**) Condition-specific participant average confusion matrices and decoding performances (i.e., accuracy) yielded during GO blocks across the IPS, the LPFC, the SMC and the M1. There were total of 3960 samples per comparison given that there were 11 participants, 6 GO trials, 3 off-trials per on-trial, 10 time-points (fMRI volumes) and 2 classes (i.e., on and off-trials) across the GO block. However, each time point is not independent from each other. The green squares denote instances where the decoding performances were significantly greater during the GO block compared to the REST block.

To demonstrate that the information of imagining specific movements could be decoded from the BOLD states yielded during the BCI diagnostic task, we classified the *on-trials* against each of the *off-trials* for each imagined movement conditions using a shifting-windows approach (see Materials and Methods). Then, the decoding performances yielded during the imagining were compared against that during the rest across the participants. It was expected that decoding performance should be greater during the imagining (i.e., GO blocks) compared to rest (i.e., REST blocks; Fig. 2a).

The results revealed that classification accuracy was significantly greater (Wilcoxon rank sum tests; all *p* < 0.0001) during the GO blocks than the REST blocks (Fig. 2b). The condition-specific, participant average decoding performance yielded during the GO and REST blocks, can be found in Table 1. Note, that a more conservative chance-level derived using a binomial cumulative distribution^19^ with a sample size of 198 (11 participants × 6 trials × 3 off-trial comparisons) is 56.06% at *p* < 0.05 (58.08% at *p* < 0.01 and 61.11% at *p* < 0.001). All participant-average decoding performance during the GO blocks were above the chance-level and that during the REST blocks were below or at the chance-level (at *p* < 0.05; Table 1).

**Table 1:** BCI-diagnostic task condition-specific participant-average decoding performance.

Reg	Decoding performance ± standard deviation %							
	Imagined movement conditions							
	LA		RA		WF		LB	
	GO	Rest	GO	Rest	GO	Rest	GO	Rest
**IPS**	60 ± 33	51 ± 33	60 ± 33	48 ± 34	64 ± 32	51 ± 34	65 ± 31	56 ± 34
**LPFC**	61 ± 33	49 ± 33	59 ± 33	48 ± 33	66 ± 30	50 ± 33	63 ± 32	53 ± 34
**SMC**	62 ± 31	48 ± 34	57 ± 34	48 ± 33	62 ± 32	50 ± 33	63 ± 32	52 ± 34
**M1**	67 ± 29	55 ± 34	60 ± 31	52 ± 33	65 ± 30	51 ± 35	66 ± 30	52 ± 34

Reg: region; LA: left ankle; RA: right ankle; WF: walk forward; LB: lean back.

**Figure 3:**
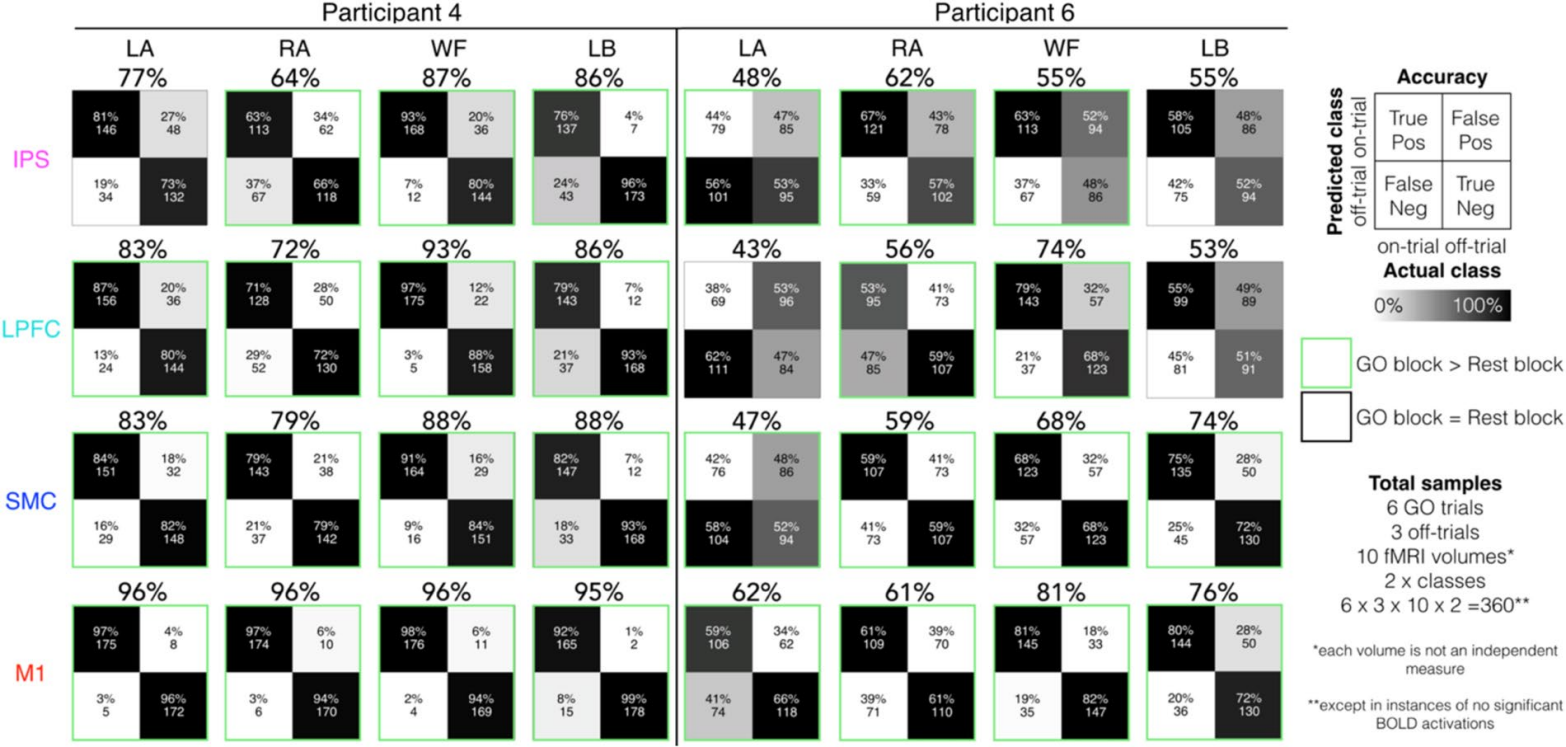
Participant-specific unimodal classification decoding performance during the BCI diagnostic task. Specific imagined movement of the conditions, left ankle, right ankle, walk forward, and lean back, could be decoded against each other at varying levels across different participants. Individual-level confusion matrices and decoding performances (i.e., accuracy) yielded during the GO blocks for each condition and region for representative participants 4 and 6. The data for all participants can be found in Supplementary Fig. 1. Because there were 6 GO trials, 3 off-trials comparisons per on-trial, 10 fMRI volumes and 2 classes (i.e., on and off-trials), there were a total of 360 samples, except in instances where no significant activations were found. Each fMRI volume time-point is not considered as an independent measure. The green squares denote instances where the decoding performances were significantly greater during the GO block compared to the REST block.

At the individual-level, imagined movements could be decoded in at least one region of the dorsal motor network for most participants (10/11 participants; Wilcoxon rank sum tests; all *p* ≤ 0.0220). Furthermore, high spatiotemporal resolution 7T-fMRI was sensitive to the intersubjective variability of decoding performances (Fig. 3; all participant data in Supplementary Fig. 1).

### Multimodal classification improves decoding performance of specific imagined movements

We then investigated whether combining the information from multiple regions could improve the decoding performance. The results revealed that for each imagined movement condition, adding information from the M1 to other areas increased the decoding performance (i.e., bimodal classification with IPS + M1, LPFC + M1, and SMC + M1 compared with unimodal classification with IPS, LPFC, and SMC, respectively; all *p* ≤ 0.0242; see Supplementary Fig. 2 for the decoding performances of all bimodal classification combinations). The condition-mean decoding performance were; 65 ± 13% > 62 ± 10% (IPS + M1 > IPS), 65 ± 14% > 62 ± 11% (LPFC + M1 > LPFC) and 65 ± 13% > 61 ± 10% (SMC + M1 > M1).

**Figure 4:**
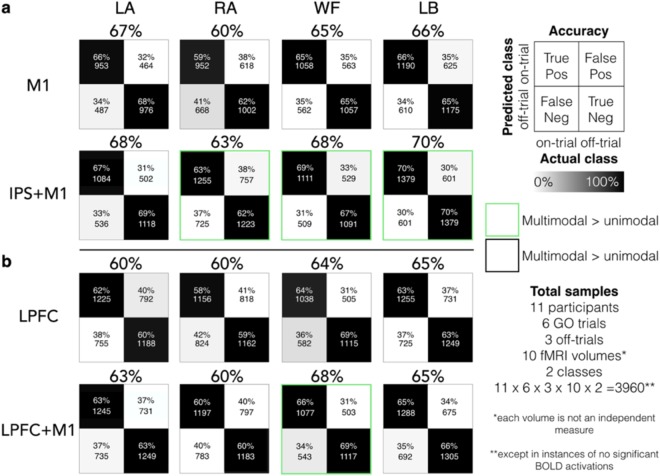
Comparison between unimodal and bimodal classification approach. Bimodal classification improved decoding performance compared to unimodal classification (see Supplementary Fig. 2 for the decoding performances of all bimodal classification combinations). Condition-specific participant average confusion matrices and decoding performances yielded during GO blocks across specific bimodal combination of the dorsal motor network regions. There were total of 3960 samples per comparison given that there were 11 participants, 6 GO trials, 3 off-trials per on-trial, 10 time-points (fMRI volumes) and 2 classes (i.e., on and off-trial) across the GO block. However, each time point is not independent from each other. The green squares denote instances where the decoding performances were significantly greater during the GO block compared to the REST block.

Furthermore, the results revealed that adding information from the IPS to the M1 marginally improved the decoding performance compared to unimodal classification with the M1 information alone for 3 of 4 conditions (Fig. 4a). The condition-mean decoding performance across the participants were 65 ± 13% > 63 ± 14% (M1 + IPS > M1). Adding information from the LPFC to the M1 marginally improved the decoding performance compared to unimodal classification with M1, only in 1 of 4 conditions (65 ± 14% > 63 ± 14%; M1 + LPFC > M1; all *p* ≤ 0.0242; Fig. 4b). Adding information from the SMC to the M1 did not improve the decoding performance (Supplementary Fig. 2).

Adding information from three regions (e.g., IPS + M1 + SMC vs IPS + M1 or LPFC + M1 + SMC vs LPFC + M1) did not improve the decoding performance compared to bimodal classification with IPS + M1 and LPFC + M1 in any of the conditions. Adding information from all areas (i.e., multimodal classification with four regions; IPS + LPFC + SMC + M1) improved the decoding performance compared to bimodal classification with IPS + M1 (70 ± 14% > 68 ± 15%) and LPFC + M1 (70 ± 14% > 67 ± 16%; all *p *< 0.0262) only in 1 of 4 conditions.

To control for the confounding factors of visual activity, increased number of features and head motion, we investigated whether adding information from the visual cortex to each of the dorsal motor network areas would improve the decoding performance. The results revealed that the adding visual cortex information did not improve the decoding performance. In fact, unimodal classification with the dorsal motor network areas yielded better decoding performances (condition and participant decoding performance: 62 ± 10% >± 54 ± 11%, IPS > visual cortex + IPS); 61 ± 11% >± 54 ± 10%, LPFC > visual cortex + LPFC; 60 ± 10% >± 55 ± 11%, SMC > visual cortex + SMC; and 63 ± 14% >± 54 ± 10%, M1 > visual cortex + M1; all *p* < 0.0033).

### Systematic unimodal and multimodal classification can identify the ideal combination of regions for classifying imagined movements

We then investigated whether systematically performing unimodal and multimodal classification could reveal the ideal region(s) for classifying imagined movements at the individual-level. The decoding performance values yielded across all conditions, trials, off-trial comparisons and GO block time-points were compared across all possible combination of dorsal motor network regions. The results revealed that for 8 out of 11 participants, ideal region(s) that yielded the best decoding performance could be identified, where significant differences between the possible combinations were revealed (Table 2).

**Table 2:** Condition average unimodal classification decoding performance, and the ideal combination of imagined movement decoding.

Participants	Trial, off-trial comparison, and time-point mean decoding performance ± standard error% averaged across the conditions)				
	Unimodal				
	IPS	LPFC	SMC	M1	
**1**	57 ± 2	57 ± 2	57 ± 1	56 ± 4	
**2**	84 ± 2	80 ± 3	73 ± 1	78 ± 4	
**3**	59 ± 1	56 ± 2	57 ± 2	52 ± 2	
**4**	79 ± 5	83 ± 4	84 ± 2	96 ± 0	
**5**	55 ± 3	47 ± 1	54 ± 3	50 ± 1	
**6**	55 ± 2	56 ± 6	62 ± 5	70 ± 4	
**7**	61 ± 2	61 ± 2	56 ± 2	52 ± 2	
**8**	61 ± 3	57 ± 2	55 ± 2	56 ± 1	
**9**	62 ± 4	58 ± 4	51 ± 3	52 ± 2	
**10**	60 ± 2	69 ± 2	67 ± 4	74 ± 7	
**11**	48 ± 0	51 ± 2	52 ± 0	56 ± 0	
**Participants**	**Region or regions (trial, off-trial comparison, and time-point mean decoding performance ± standard error% averaged across the conditions)**				***p*** **≤** ^*^
**1**	—				—
**2**	IPS (84 ± 2)				0.0109
**3**	IPS (59 ± 1) or LPFC (56 ± 2) or SMC (57 ± 2)				0.0016
**4**	M1 (96 ± 0)				0.0163
**5**	IPS (55 ± 3) or SMC (54 ± 3) or M1(50 ± 3)				0.0014
**6**	M1 (70 ± 4)				0.0167
**7**	IPS (61 ± 2) or LPFC (61 ± 2)				0.0057
**8**	IPS (61 ± 3)				0.0056
**9**	IPS (62 ± 4)				0.0092
**10**	LPFC+M1 (80 ± 4) or SMC+M1 (77 ± 5)				0.0134
**11**	—				—

- : Instances of no significant differences between the different region combinations; ^*^p-value thresholds are False Discovery Rate adjusted.

### Decoding performance from the BCI diagnostic task predicts the performance during the BCI simulation task

Lastly, we investigated whether a given individual’s decoding performance yielded during the BCI diagnostic task could predict that yielded during the BCI simulation task. During the BCI simulation task, significant BOLD activity was observed during imagining of specific movements for most participants in the IPS (10/11 participants; 1611 ± 396 mm^3^), in the LPFC (9/11 participants; 1044 ± 272 mm^3^), in the SMC (7/11 participants; 456 ± 138 mm^3^) and the dorsomedial M1 (7/11 participants; 336 ± 95 mm^3^). Each imagined movement condition could also be classified from the BOLD signals. The decoding performance was expected to be greater during the second half of the trial block compared to the first half during the BCI simulation task, due to the hemodynamic response delay and the lack of prompt and REST blocks (see Statistics under Materials and Methods for a more detailed explanation and examples). Consistently, average decoding performance was significantly greater during the second half of the trial than the first half for all conditions in all major areas of the dorsal motor network (condition-mean decoding performance ± SD across participants; IPS, 58 ± 5% >52 ± 4%; LPFC, 57 ± 6% >52 ± 5%; SMC, 58 ± 6% > 51 ± 3%; and M1, 62 ± 11%>54 ± 4%; Wilcoxon rank sum tests; all *p* ≤ 0.0004; Table 3).

**Table 3:** Condition-specific trial-averaged decoding performance and the dice’s coefficients for each individual in the primary motor cortex.

Par	Trial-averaged decoding performance ± standard error and dice’s similarity coefficient values %											
	Imagined movement conditions											
	LA			RA			WF			LB		
	Diag	Simul	Dice	Diag	Simul	Dice	Diag	Simul	Dice	Diag	Simul	Dice
**1**	66 ± 2	64 ± 2	32	59 ± 2	61 ± 2	12	47 ± 3	65 ± 2	4	53 ± 3	55 ± 2	0
**2**	69 ± 2	—	0	—	60 ± 2	0	76 ± 2	54 ± 2	6	89 ± 2	—	0
**3**	46 ± 3	—	0	57 ± 3	—	0	51 ± 3	—	0	53 ± 3	—	0
**4**	96 ± 1	95 ± 1	31	96 ± 1	95 ± 1	33	96 ± 1	84 ± 2	25	95 ± 1	80 ± 2	15
**5**	—	—	0	52 ± 2	—	0	—	—	0	49 ± 3	—	0
**6**	62 ± 2	67 ± 2	7	61 ± 3	80 ± 2	18	81 ± 2	74 ± 2	45	76 ± 2	63 ± 2	0
**7**	—	50 ± 2	0	—	57 ± 2	0	56 ± 2	58 ± 2	0	49 ± 2	49 ± 2	5
**8**	55 ± 2	—	0	60 ± 2	—	0	53 ± 2	54 ± 2	5	58 ± 2	—	0
**9**	51 ± 2	51 ± 2	12	48 ± 2	46 ± 2	0	59 ± 3	56 ± 2	39	52 ±3	52 ± 2	0
**10**	89 ± 2	75 ± 2	34	54 ± 3	74 ± 2	0	71 ± 2	54 ± 2	5	83 ± 2	70 ± 2	3
**11**	—	55 ± 2	0	56 ± 3	52 ± 2	6	—	69 ± 2	0	—	51 ± 2	0

Par: participants; Diag: BCI diagnostic task; Simul: BCI simulation task; —: Instances of no significant BOLD activation.

Dice: Dice’s coefficient that measures the percentage of overlap of BOLD activation maps between Diag and Simul.

**Figure 5:**
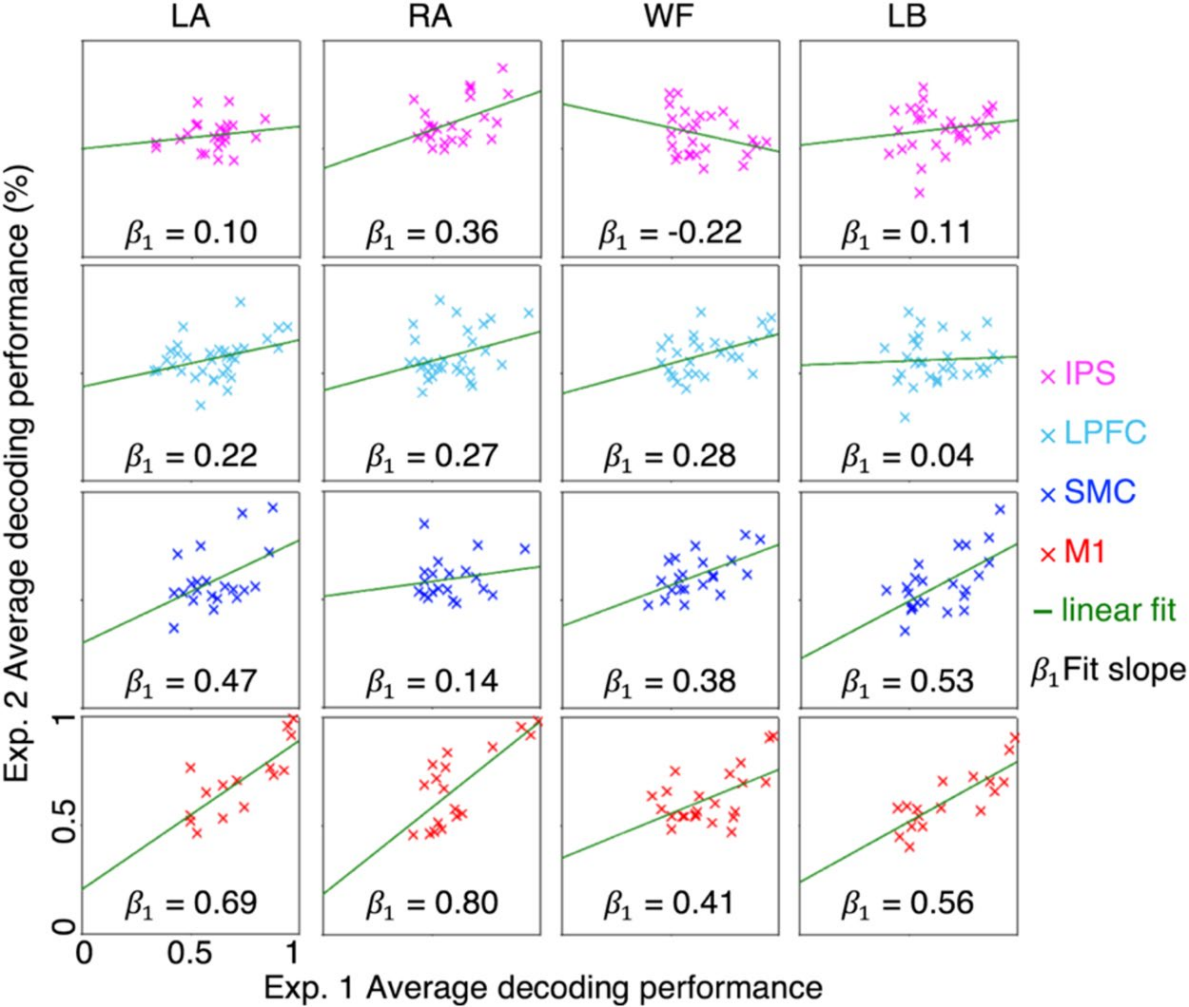
BCI performance prediction. Each figure plots the average decoding performance during the GO blocks in BCI diagnostic task versus that during the second half of the experimental block in BCI simulation task. Each column denotes the conditions, each column/color denotes the region in which the unimodal classification was conducted.

Linear regression analyses revealed that the decoding performance yielded during the BCI diagnostic task could significantly predict the decoding performance yielded during the BCI simulation task in the M1 for all four conditions (LA: *F*_(1,13)_ = 21.51, *R*^2^ = 0.62, *p* < 0.0005, 95% confidence interval (CI) [0.37, 1.00]; RA: *F*_(1,16)_ = 21.40, *R*^2^ = 0.57, *p* < 0.0003, 95% CI [0.43, 1.116]; WF: *F*_(1,22)_ = 9.78, *R*^2^ = 0.31, *p* < 0.0049, 95% CI [0.14, 0.68]; and LB: *F*_(1,16)_ = 31.93, *R*^2^ = 0.67, *p* < 0.0001, 95% CI [0.35, 0.76]; Fig. 5).

Subsequently, we quantified each individual’s ability to generate consistent spatial patterns of BOLD activation across the tasks in the M1 by calculating Dice coefficients (Table 3). Supportive of the notion that the BCI diagnostic task could potentially estimate one’s ability to control BCIs, linear regression analysis revealed that the variance across the participants’ decoding performances during the BCI simulation task could be significantly explained by the variance across the condition-averaged Dice coefficients (*F*_(1,9)_ = 26.84, *R*^2^ = 0.75, *p* < 0.0006, 95% CI [0.65, 1.65], *r* = 0.87).

## Discussion

We demonstrate the feasibility of identifying the ideal location(s) for classifying mental strategies using high spatiotemporal resolution 7T-fMRI. Cortical regions underlying imagination of specific movements could be identified at the individual-level (Fig. 1), and each imagined movement could be classified based on the BOLD states across the population (Fig. 2) and at the individual-level (Fig. 3; Supplementary Fig. 1). Multimodal classification improved the decoding performance compared to unimodal classification, suggesting that 7T-fMRI is sensitive to the differences in the information being processed across the dorsal motor network areas. And further, it suggests that utilizing different information across different areas could potentially improve the decoding performance (Fig. 4). The use of both unimodal and multimodal classification approach revealed the ideal combination of regions for classifying imagined movements at the individual-level (Table 2). Lastly, the participants’ decoding performances during the BCI diagnostic task significantly predicted the performances during the BCI simulation task (Fig. 5).

A potential application of this work may be to pre-surgically determine the ideal electrode implantation sites for BCI control based on the functional neuroimaging data – if indeed the regions of BOLD activation are localized to the sites of underlying neural activity. Previous efforts to utilize fMRI as a pre-surgical tool relied on clinical fMRI^1^, where a substantial proportion of the BOLD signal arises from distant draining veins away from the sites of neural activity^20–22^, decreasing the specificity of the BOLD activation maps produced. However, the extent of such non-local BOLD activation decreases as the magnetic field strength increases, which in turn, increases the specificity of the BOLD activation to the underlying neural activity^10,23,24^. At ultra-high magnetic field strength of 7T, the rate of signal decay (i.e., T_2_^*^) from the large veins is extremely short (i.e., ~7 ms)^10,11^. By the time the signal is sampled, the level of BOLD signal contribution is substantially diminished^15,25–28^. Consequently, the current use of high spatiotemporal resolution 7T-fMRI provides a level of confidence that the sites of BOLD activation observed for each imagined movement condition and the corresponding information decoded are localized to the source of underlying neural activity. Supportively, 7T-fMRI BOLD signals and population neural activity measured during a digit motor-mapping task have shown substantial somatotopic congruency, where the activation foci of individual fingers were matched at the millimeter scale at the individual-level^15^. Being able to pre-surgically identify ideal areas for information decoding may be especially useful for invasive BCIs, as the implantation procedures require general anesthesia and the candidate patients are not healthy enough to undergo an awake neurosurgery for electrical stimulation brain-mapping.

Consistent with the notion that the pattern of 7T-fMRI BOLD activations may be localized to the underlying neural substrates, BOLD states yielded during each imagined movement could be classified from one another across all dorsal motor network areas. In addition, the results suggest that different regions of the dorsal motor network process different types of task-relevant information, and importantly, the information being processed in some regions may be more characteristic to the mental strategies than others. Thus, utilizing information from a specific set of regions could lead to better characterization of imagined movements compared to unimodal classification. For example, the current results revealed that decoding performance was not only greater with multimodal classification compared to unimodal classification approach, but, the best decoding performance with the least number of regions was achieved when the information from the IPS and the M1 were used. Fittingly, the IPS has been implicated in multisensory information integration^29^, motor planning^30–32^, working memory^33^ and specific motor goal-encoding^34,35^. It is of general scientific consensus that the M1 generates the majority of afferent signals that ultimately engage the skeletal muscles for movements, and that it is somatotopically organized. Also, studies have shown that specific imagined movements can lead to putative somatotopic activations in the M1^36–40^. Indeed, both the IPS^5^ and the M1^6,18,41–43^, each have been the site of electrode implantation in previous demonstrations of restorative-BCIs.

The results also suggest that 7T-fMRI is sensitive to the potential variabilities in cortical functional organisation across the participants. For example, robust patterns of BOLD activity across the dorsal motor network areas were observed for participant 4, while participant 11 failed to generate any significant BOLD activation for some of the imagined movement conditions. Consistently, for participant 4, imagined movements could be classified against each other based on the BOLD states with 95% accuracy on average in the M1, while none could be decoded above chance for participant 11 (Supplementary Fig. 1). The inter-subject variability is consistent with the large SD of the group-average decoding performance, and also accounts for the rather low levels of group-mean decoding performance values (all participant-average decoding performance were above the conservative chance-level calculated with binomial cumulative distribution at *p* < 0.05; Table 1). Furthermore, by classifying the imagined movements using both unimodal and multimodal classification approach, we were able to identify the specific region(s) that yielded the best decoding performance with the least number of areas (Table 2). These results suggest that 7T-fMRI could be utilized to identify the ideal sites for mental strategy classification prior to surgery, which could potentially be used to improve the electrode array placements for future BCI treatments.

However, whether a given individual’s decoding performance achieved during the current 7T-fMRI protocol is predictive of one’s ability to control BCIs in real-life cannot be proven until the system is implemented. Thus, to provide at least some measure of comparison, the participants engaged in the BCI simulation task. The task attempted to artificially induce a facet of neural states that reflects BCI-operation in a real-life scenario, where the user not only has to generate but consecutively switch between distinct neural states. Our results showed that the decoding performances during the BCI simulation task could be significantly predicted by the BCI diagnostic task, providing some support 7T-fMRI could be utilized as a pre-surgical BCI diagnostic tool. In addition, the decoding performance during the BCI simulation task could also be predicted from the Dice coefficient values (i.e., extent of spatial overlap in BOLD activation patterns across the two tasks), suggesting that individuals who achieved better decoding performances may be better at eliciting a more consistent pattern of BOLD activity across different situations. However, the Dice coefficient values were relatively low, even for well-performing individuals, which may have been due to the differences between the two tasks demanding slightly different cognitive states during the imagined movements. For example, the BCI diagnostic task was inherently more complex compared to the BCI simulation task, as the participants had to plan an imagined movement, perceive a cue, interpret it, then decide to inhibit or engage in the planned mental strategy. On the other hand, the participants were always aware that the mental strategies were to be performed in the BCI simulation task.

We acknowledge that the decoding performance achieved during BCI simulation task is not an adequate measure to infer one’s ability to control BCIs decisively. However, it is something that can only be quantified retrospectively. Thus, future BCI studies with ethical approval should attempt to update the pre-surgical screening protocols according to the currently available fMRI methods to provide the field with the invaluable data to better investigate whether some pre-surgical procedures can predict the efficacy of BCIs at the individual-level.

### Caveats

To note, previous motor cognition studies involving lower-limb movement execution^44^ and observation^39^ have reported robust activations in the superior parietal lobule (SPL), but not in the current study. Mostly weak SPL activation was sporadically observed, unlike the other areas that showed robust activation across the conditions and participants. It may be that the use of imagined movements and/or the differences in the experimental paradigm between previous studies and the current resulted in the lack of SPL activation. For example, a study by Hotz-Boendermaker *et al*.^45^ reported BOLD activations in the parietal areas during motor imagery in healthy individuals to be focused in the IPS but not in the SPL. A study by Heed *et al*.^44^ reported robust SPL activity during foot movements, however, the motor tasks involved spatial memory (i.e., encoding and navigating to the target location), which is often attributed to many regions of the SPL^46^.

The pattern of neural activity may be different during imagining movements inside an MRI environment compared to during a real-life BCI-control scenario. During the latter, additional information of one’s performance will be fed back to the user. The differences in neural activity patterns that are driven by such situational nuances may hinder the predictability of the current diagnostic task. However, our results suggest that, although some differences in neural states may indeed be induced when performing the same mental strategy in different situations (e.g., BCI diagnostic task *vs* BCI simulation task), common information can be decoded and be predictive intra-subjectively. Furthermore, it has been shown that performance feedback can actually induce neuroplasticity in a way that favors the BCI performance outcomes^47^, which may potentially minimize the differences in neural states induced by situational nuances over time.

Unlike the paralyzed individuals, healthy individuals may have to actively inhibit the movements during motor imagery. This could have induced some motion artifacts in correlation with the time-points of imagination in the current study. However, we controlled for the motion artifacts biasing the results by identifying the time-points of significant movements and adding them as nuisance variables in the GLM, on top of performing motion-correction^48^. The results suggested that the observed effects were not due to motion (and visual) artifacts, as adding information from the visual cortex did not improve the decoding performance compared to unimodal classification with each of the dorsal motor network areas. In fact, the decoding performance was better with unimodal classification (the notion here is that significant and correlated motion artifacts should be observed globally and most prevalently in the edges of the brain; e.g., visual cortex).

The potential cognitive/neural differences during internalized motor mental strategies between the paralyzed and non-paralyzed population may also influence the decoding performances. While some nuances in the neural substrates have been described between the two populations during a variety of internalized motor cognitions^45,49^, studies have also shown that there is an extensive overlap in the neural substrates all involving the dorsal motor network^45,50–53^. In addition, the BCI diagnostic task is indifferent to the different types of internalized motor mental strategies or paralysis status of the individual, as its purpose is to identify the neural substrates that correspond to the intent to engage in some internalized cognition. The delayed prompt blocks ensure that the participants are well aware of the task to perform, and that the cognitive processes involved in imagination of specific movements can be isolated from the planning processes. The eyes are to be fixated on the center-cross at all times and the stimuli are visually matched exactly between the prompt, the GO and the NO-GO blocks, except for the colour to induce as little discrepancies. Contrasting the BOLD states between the GO and the NO-GO trials provides a level of confidence that the intent of engaging in the mental strategy is captured. Thus, the same task can be utilized in paralyzed individuals while they either imagine or attempt to perform specific movements, then the locations of BOLD activity and the ideal regions for information classification can be identified accordingly. Imagined movements were simply used in this study, given the constraint of only being able to recruit healthy, non-paralyzed individuals.

## Conclusion

The current work demonstrates the feasibility of utilizing 7T-fMRI with unimodal and multimodal classification for localizing ideal sites of mental strategy classification. The results show that high spatiotemporal resolution 7T-fMRI can detect robust BOLD activations corresponding to specific internalized motor cognitions across the long-range dorsal motor network, and unimodal and multimodal classification can reveal the ideal location(s) for classifying the information at the individual-level. Furthermore, the participants’ decoding performances during the BCI diagnostic task could predict that during the BCI simulation task.

## Materials and Methods

### Participants

Eleven healthy volunteers (5 males and 6 females; mean ± standard deviation age: 25±5 years) participated in a single-session fMRI experiment. Each participant gave informed consent prior to their participation. The data was anonymized before the analyses. The University of Melbourne Human Ethics Committee approved this study (Ethics ID: 1340926.1). All experiments were performed in accordance with relevant guidelines and regulations.

### Behavioral protocol

#### BCI diagnostic task

**Figure 6:**
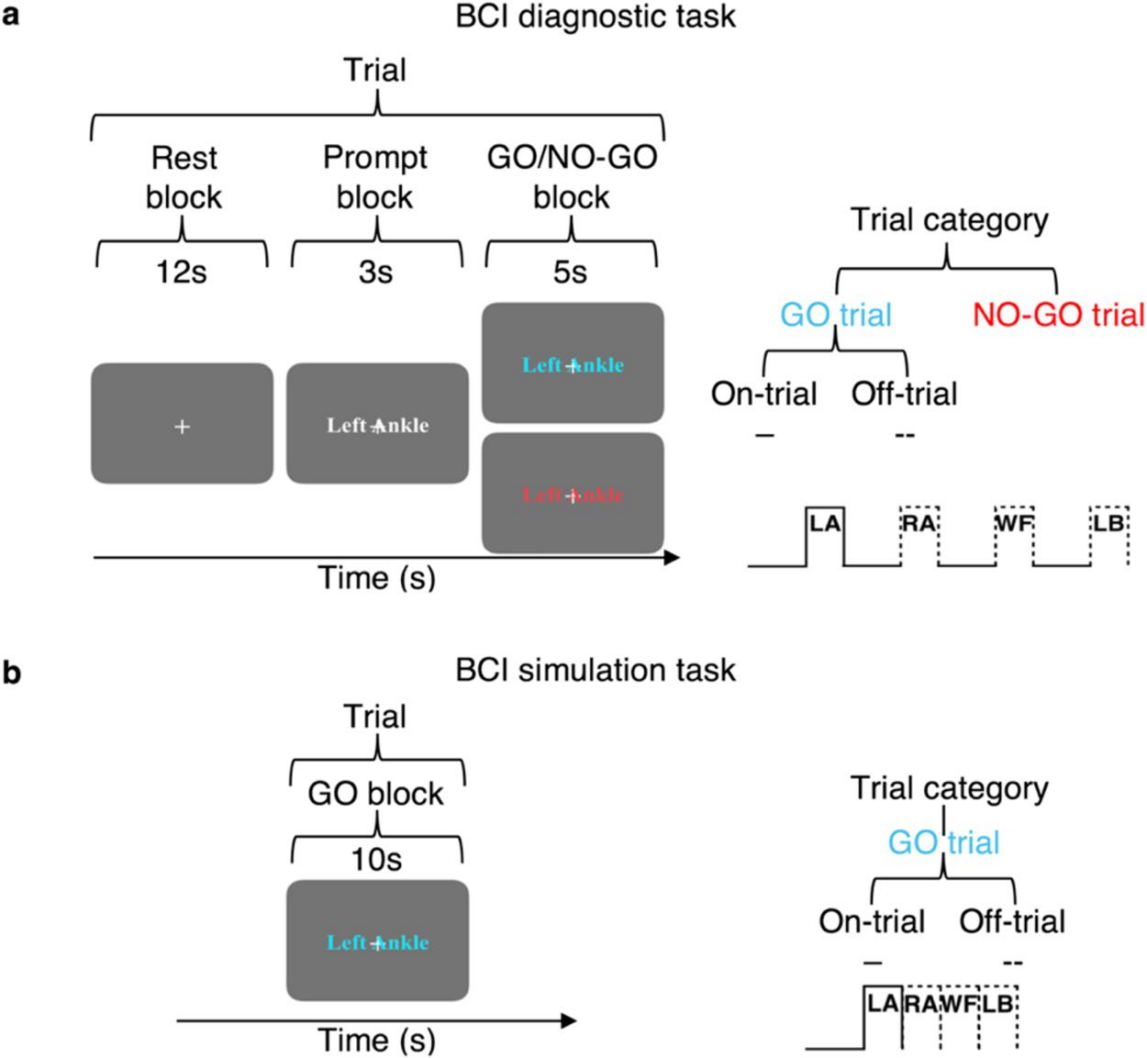
Experimental designs of the BCI diagnostic task and BCI simulation task and the conventions used to describe specific trial types. (**a**) Each trial of the BCI diagnostic task consisted of 12s of REST block, 3s of prompt block then a 5s GO or NO-GO block. Each trial can either be a GO or a NO-GO trial (75:25 ratio). Total experimental time was 10min 57s (**b**) Each trial of the BCI simulation task consisted of 10s of GO block. There were four imagined movement conditions, and the trial category was randomly chosen with equal chance at each trial repetition. Total experimental time was 5min 37s. For a given set of significant voxels that modelled a particular condition, the corresponding trial blocks of that condition is referred to as on-trial, and the rest are referred to as off-trials. An example of on and off-trials for the significant voxels for the left ankle condition is shown on the bottom right figures of a and b.

In the BCI diagnostic task, we investigated whether the information of imagining specific movements can be isolated *via* and decoded from BOLD signals. Participants followed the instructions on the screen (Fig. 6a). Before the task began, the words “ARE YOU READY?” were presented for 5s. An experimental trial lasted for 20 s, consisting of three separate blocks: a 12 s REST block with a white fixation cross in the center of the screen; a 3 s prompt block with a visual cue, “Left Ankle” (LA), “Right Ankle” (RA), “Walk Forward” (WF) or “Lean Back” (LB), overlaid onto the fixation cross indicating movement was to be imagined; then a 5 s GO/NO-GO block was presented where the colour of the visual cue either changed to cyan or red, respectively. If the visual cue turned cyan the participants were to imagine the corresponding movement (i.e., GO trial), and if it turned red, the participants were to cancel the imagined movement and resort back to rest (i.e., NO-GO trial). The four conditions, LA, RA, WF and LB were randomly presented. Each GO trial condition was repeated 6 times, and NO-GO trial was repeated 2 times (prevalence ratio of GO:NO-GO trials, 75:25). All experiments finished with 12 s of REST block. Thus, a given trial lasted for 20 s, there was a total of 32 trials (24 GO trials and 8 NO-GO trials), and inclusive of the initial 5 s ready and the final 12 s rest period, the entire task lasted for 10 min and 57 s.

Participants were instructed to imagine performing the movements by concentrating on the feeling of the movements and to visualize the movements if desired, with their eyes fixated on the fixation cross at all times. It was specifically emphasized that the participants should imagine the movements in first-person point-of-view, and to keep still at all times. For LA and RA, the participants were instructed to imagine performing ankle plantarflexions at the rate of 1-flexion per second. For WF, the participants were instructed to imagine themselves walking forward towards the screen at the rate of 2-steps per second. For LB, the participants were instructed to imagine shifting their bodyweight backwards against the MRI bed continuously.

#### BCI simulation task

To provide at least some measure of real-life BCI operation performance as a comparison, we attempted to simulate the cognitive events that may take place when the users try to control BCIs in a real-life scenario. Namely, where the user not only has to generate, but consecutively switch between distinct neural states using different mental strategies to generate a sequence of input commands (Fig. 6b). Before the task began, the words “ARE YOU READY?” were presented for 5 s. An experimental block consisted of 10s of imagining the movements, where a cyan visual cue, “Left Ankle”, “Right Ankle”, “Walk Forward” or “Lean Back” were overlaid onto a fixation cross. The four conditions were randomly presented. Each condition was repeated 8 times. All experiments finished with 12 s of REST block. The participants were instructed to perform the imagined movements using the identical strategies as in the BCI diagnostic task. Thus, a given trial lasted for 20 s, there was a total of 32 trials, and inclusive of the initial 5 s ready and the final 12s rest period, the entire task lasted for 5 min 37 s.

#### Terminology

For the signal time-series extracted from a set of sub-regions (voxels) showing significant BOLD activation during imagination of a specific movement, the time-points when the corresponding movements were imagined are referred to as *on-trials*, and all other trials as *off-trials*. This terminology can be best understood through an example. Consider a set of sub-regions that activated significantly during imagining of left ankle movements (i.e., LA GO>NO-GO). For these specific set of sub-regions, the time-points when LA was imagined are referred to as on-trials. On the other hand, the time-points when RA, WF or LB was imagined are all referred to as off-trials (Fig. 6). By that notion, for the set of sub-regions that activated significantly during imagining of right ankle movements, the RA trial blocks are referred to as the on-trials, while the LA, WF, and LB trial blocks are referred as the off-trials, and so on.

### fMRI protocol

#### Image acquisition

All imaging was performed on a 7T research scanner (Siemens Healthcare, Erlangen, Germany) with a 32-channel head-coil (Nova Medical Inc., Wilmington MA, USA). Whole-brain high-resolution T_1_-weighted anatomical images were acquired for each participant using magnetization-prepared rapid gradient echo sequence (MP2RAGE; voxel volume = 0.9 × 0.9 × 0.9 mm^3^; iPAT factor = 4; TR = 4900 ms; transmitter voltage (TX)=240V). All fMRI images were acquired using 2D gradient echo-echo planar imaging (GE-EPI) with multiband and parallel imaging acceleration (Siemens Healthcare prototype research sequence) with the following acquisition parameters: bandwidth=1690Hz/pixel; echo time = 24 ms; repetition time = 500 ms; echo spacing = 0.74 ms; EPI factor = 148; phase encoding shift factor = 2; voxel volume = 1.5 × 1.5 × 1.5 mm^3^; in-plane field of view (FOV) = 222 × 222 mm^2^; flip angle = 34^o^, where T_1_ = 2000 ms; partial Fourier = 6/8; phase encoding direction = A-P; multiband factor = 3; GRAPPA factor = 3; number of slices = 27; slice FOV = 40.5 mm. Two additional sets of GE-EPI images with opposing phase encoding directions were acquired to perform B_0_-distortion correction.

#### Image analysis

We used a customized analysis pipeline due to the partial-coverage 7T-fMRI data (i.e., *optimized partial-coverage functional analysis pipeline OPFAP*^54^). OPFAP consists of many steps and sub-pipelines, thus, the details are beyond the scope of this study. We recommend referring to our previous work for technical information^54^.

The FMRIB’s Software Library’s (FSL v6.0) was used for functional analyses^48^. The two additional sets of GE-EPI images with opposing phase-encoding directions were used to estimate and correct susceptibility-induced off-resonance field using FSL’s *top-up* and *applytopup* functions^48^. Then, the distortion-corrected functional images were motion corrected, high-pass filtered (0.01Hz), skull-stripped, but were not smoothed. No slice-timing corrections were employed given the fast TRs and multiband acceleration.

Individual-level fMRI analyses were carried out in the following steps. To delineate the voxels in regions corresponding to the planning of imagined movements from the actual imagining, the prompt, GO and NO-GO blocks were modelled as separate box-car functions. Box-car functions were convoluted with a gamma function (mean = 6s, standard deviation = 3s) then were used as predictor variables for multiple regression analysis. Furthermore, the time-points of significant movements identified during the motion-correction step were included into the model as nuisance variables, in order to further control for the motion artifacts. The resulting *Z-*score maps of the GO trials were contrasted against the corresponding NO-GO trials (e.g., LA GO>LA NO-GO). Significant activation was defined using a lower *Z-*score threshold of 2.3 (with *p* < 0.05 for significance testing; cluster-based correction).

The individual-level activation maps were masked with the participant-specific ROI masks to ensure fair comparisons of decoding performances within the dorsal motor network regions across participants. The ROIs were manually defined in a study-specific template brain space, then were transformed into each participant’s functional space to avoid potential human error or bias of defining inconsistent ROIs across individuals. We opted to define the ROIs manually, instead of utilizing atlas-based approach, as the partial-coverage GE-EPI images acquired at 7T did not co-register well with FSL’s default standard brain space, Montreal Neurological Institute space (e.g., MNI_152)^54^.

The ROI masks for the major dorsal motor network areas IPS, the LPFC, the SMC and the M1, and also the visual cortex, were created in the following steps. First, a study-specific template brain was created using all T_1_-weighted anatomical images using the Advanced Normalization Tools (ANTs)^55^. Note that during the creation of study-specific template brain, the affine transformation and deformation parameters from template brain space to each individual’s anatomical space were automatically calculated and saved (i.e., anatomical space ←→ template space). Second, each participant’s center time-point of the fMRI image series (i.e., motion-correction reference image) was linearly registered to the corresponding T_1_-weighted anatomical images using FSL’s LInear Registration Tool (FLIRT) with boundary-based registration to calculate the transformation parameters from the functional space to the anatomical space (i.e., functional space → anatomical space). Third, the masks were manually drawn, slice-by-slice (axially), for the IPS, the LPFC, the SMC, the M1 and the visual cortex in the study-specific template brain. Fourth, using the inverse of the transformation and deformation parameters calculated above, these masks were transformed into the participants’ own functional image spaces in a step-wise manner (i.e., template space → anatomical space [*via* ANTs] → functional space [*via* FLIRT]). In turn, five ROI masks were created for each participant. At the individual-level, significant voxels for the various contrasts were masked with each of the ROI masks (see Supplementary Fig. 3 for functional-to-template space co-registration results of all participants).

#### BOLD activation percent signal change calculation and time-course extraction

The BOLD activation percent signal change (%∆*S*) time-series was calculated in the following way. For each voxel, the data were normalized by subtracting the temporal mean signal then dividing by the temporal mean signal at each time-point. The normalized BOLD time-course from each voxel of encompassing the regions of significant activation was extracted using the command *fslmeants* at the individual-level.

### Statistics

In cases of multiple comparisons, the *p*-values were tested against the False-Discovery Rate (FDR) adjusted threshold using the Benjamini-Hochberg procedure at Q = 0.05. The original *p*-values and the FDR-adjusted threshold values are reported in the text.

#### Linear classification of spatiotemporal dynamics of BOLD activation

We investigated whether the intent of imagining specific movements can be decoded from the BOLD signals recorded during the BCI diagnostic task by classifying the on-trials from each of the off-trials (see **Terminology** and Fig. 6). If indeed a given set of sub-regions underlie the imagining of a specific type of movement, then the decoding performance should be greater during the GO blocks compared to the REST blocks. For example, for the regions that activated significantly during the imagination of left ankle movements (i.e., LA GO>NO-GO), the BOLD activity should be at baseline during the REST blocks of both the left ankle trials (i.e., on-trial) and right ankle trials (i.e., off-trial), thus, the two imagined movement conditions should be indistinguishable based on the BOLD states. On the other hand, if indeed the regions encode for imagining of left ankle movements, the BOLD activity during the GO blocks should be greater in the on-trials compared to the off-trials, and thus, the two conditions should be distinguishable based on the BOLD states (Figs 2a and 6a).

For each condition, the BOLD %∆*S* time-courses from the significant voxels corresponding to the contrast of GO>NO-GO trials were subjected to linear classification using linear discriminant analysis with a with shifting-window approach (500 ms-shifting-window; i.e., every TR/sample), *via* custom script written in MATLAB (MathWorks Inc., Natick MA, USA, version R2015b). At each time-point, the classifier was trained with data from all but one GO trial blocks, with the on-trial data defined as “1” and the off-trial data defined as “0”. The classifier was then used to classify whether the on and off-trial data from the remaining GO trial was either “1” or “0”, respectively. The decoding performance was validated using a leave-one-out method, where each comparison was permutated by the number of GO trials with no repeats. Because there were 3 possible off-trial types (e.g., RA, WF and LB) for each on-trial type (e.g., LA) and a total of 6 GO trials for each imagined movement condition, 18 decoding performance values were calculated for a given condition and region at each time-point of the trial block. This process was repeated as the window shifted across time.

The classification was performed using the data from each region separately (*unimodal classification*), as well as by drawing the data from two (*bimodal classification*), three (*multimodal classification with three regions*) and four regions simultaneously (*multimodal classification with four regions*). For the decoding performance comparison between multimodal classifications, all possible combinations of the dorsal motor network regions were tested. Bimodal classifications with each dorsal motor network region and the visual cortex were also performed to investigate the potential confounding effects on the decoding performances.

#### Decoding imagined movement information from BOLD signals

To demonstrate that the information of imagining specific movements can be decoded from the BOLD signals, the decoding performance values from the BCI diagnostic task yielded during the GO block time-points (7 s–12 s after the start of prompt blocks) of all cross-validation-trials and off-trial comparisons were compared with that during the REST blocks (15 s–20 s after the start of the prompt blocks) for each region and imagined movement condition using Wilcoxon rank sum tests across the population. Thus, there was a total of 3960 samples for each comparison at the group-level (11 participants × 6 GO trials × 3 off-trials per on-trial × 10 fMRI time-points at 500 ms × 2 classes of on and off-trials). A stricter chance-level was also calculated using cumulative binomial distribution^19^ with samples size of 198, as each fMRI time-point is not independent (11 participants × 6 GO trials × 3 off-trials per on-trial).

#### Comparing decoding performance of unimodal classification and multimodal classification

We then investigated whether multimodal classification improved the decoding performance compared to unimodal classification. All decoding performance values from the BCI diagnostic task across the trials, off-trial comparisons and time-points during the GO blocks were extracted for all possible combinations of unimodal classification (number of combinations; 4), bimodal classification (6), and multimodal classification with three (4) and four regions (1) for each imagined movement condition. All decoding performance values for each classification type were compared against each other to determine whether utilizing information from more areas improved the decoding performance (one-tailed Wilcoxon rank-sum tests).

#### Identification of ideal regions for classifying imagined movements

We also investigated whether ideal regions of information classification could be identified at the individual-level. All decoding performance values from the BCI diagnostic task across the om-trials, off-trial comparisons and time-points during the GO blocks were extracted and compared across all 15 possible combinations of unimodal and multimodal classifications combinations. Then the specific combination that yielded the best classification accuracy with the least number of regions were determined, by identifying the combination where the decoding performance no longer significantly improved.

#### BCI performance prediction

Finally, to investigate whether a given individual’s performance during the BCI diagnostic task has some predictability of BCI control, we investigated whether the participants’ decoding performances during the BCI diagnostic task can predict the performances in the BCI simulation task. The regions of significant BOLD activations for each imagined movement was identified for the BCI simulation task, and the information was decoded using the same method as above. In the BCI simulation task, the decoding performance was expected to increase during the second half of a given trial compared to the first half, as the haemodynamic response delays the rise of the positive BOLD response relative to neural activation onset, which is assumed to be at the start of each trial block. Thus, to determine whether specific imagined movement execution could be decoded from the BOLD signal in the BCI simulation task, the decoding performance during the second half of the trials were compared against the first half across the population *via* Wilcoxon rank sum tests.

Then, linear regression analysis was performed between the average decoding performance values during the GO blocks in the BCI diagnostic task and the decoding performance during the second half of the experimental blocks in the BCI simulation task for each condition and classification types (unimodal and multimodal). The assumptions of multicollinearity between the predictors and the criterion, and the normality and homoscedasticity of variance were tested by examining the collinearity tolerances, the distribution of residual values on the Q-Q plot and scatter plot. Furthermore, the overlap between the regions of activation across the two tasks has also quantified for each imagined movement condition, in instances of significant BCI performance prediction with unimodal classification decoding performance data. Dice coefficients were calculated for using the formula:

$$D=\frac{2|A\cap B|}{|A|+|B|}\times 100$$

where, *D* is the Dice’s similarity coefficient in percentage and |A| and |B| are the voxel volume of the significant activation observed during the BCI diagnostic task and BCI simulation task. Linear regression analysis was also performed between the participants’ Dice’s coefficients and the BCI simulation task decoding performances.

## Electronic supplementary material

Supplementary Figures
