# Supplementary material for: Feasibility of identifying the ideal locations for motor intention decoding using unimodal and multimodal classification at 7T-fMRI
Source: Sci Rep. 2018 Oct 22;8:15556. doi: 10.1038/s41598-018-33839-4 (PMC6197258; doi:10.1038/s41598-018-33839-4)
Supplement: Supplementary file 1 — Supplementary Figures [file 41598_2018_33839_MOESM1_ESM.docx]

Supplementary information

***Feasibility of identifying the ideal locations for motor intention decoding using unimodal and multimodal classification at 7T-fMRI***

*Peter E. Yoo, Sam E. John, Nicholas L. Opie, Roger J. Ordidge, Terence J. O’Brien, Maureen A. Hagan, Thomas J. Oxley, Yan T. Wong, Bradford A. Moffat*

**Supplementary Fig. 3**. **Region-specific temporal dynamics of BOLD activation after blind-deconvolution.** Movement informaiton rose earlier in the frontoparietal regions followed by the SMC then the M. It decayed in the frontoparietal regions soon after it appeared in the SMC, while it persisted throughout movements in the SMC. LPFC and PPC had the shortest latency to peak %∆*S* followed by SMC then M1 (all *p*≤0.0411, FDR correction Q=0.1). (**a**) Left figures show the average latency to reach 50% (**top**), 80% (**middle**) and 100% (**bottom**) maximum %∆*S* from the start of the prompt block across the cortical regions. Right figures show the *p*-values of Wilcoxon rank-sum tests comparing the latency values across participants. (**b**) The participant, voxel and trial average of: %∆*S* time-course (**left**); and time-course of inverse *p*-values (i.e., 1-*p*) of Wilcoxon rank-sum tests (**right**). Significant cross-correlations with no delay were observed between %∆*S* and 1-*p* within each region (all *r*≥0.8, *p*≤0.0096). (**c**) Left plots show the average %∆*S* time-course from significant voxels. Each row represents one cortical region. The last row shows time-courses of all regions. Right plots show the average %∆*S* normalized to its relative minimum and maximum values to highlight the temporal dynamic differences across the regions. (**d**) A schematic representation of flow of movement plan, as represented by the latencies. The blue and red bars in **a** and **c** indicate the start and duration of prompt and execution blocks, respectively. The shading in **a** and **b** depict standard errors and error bars in **c** depict standard deviations across participants.


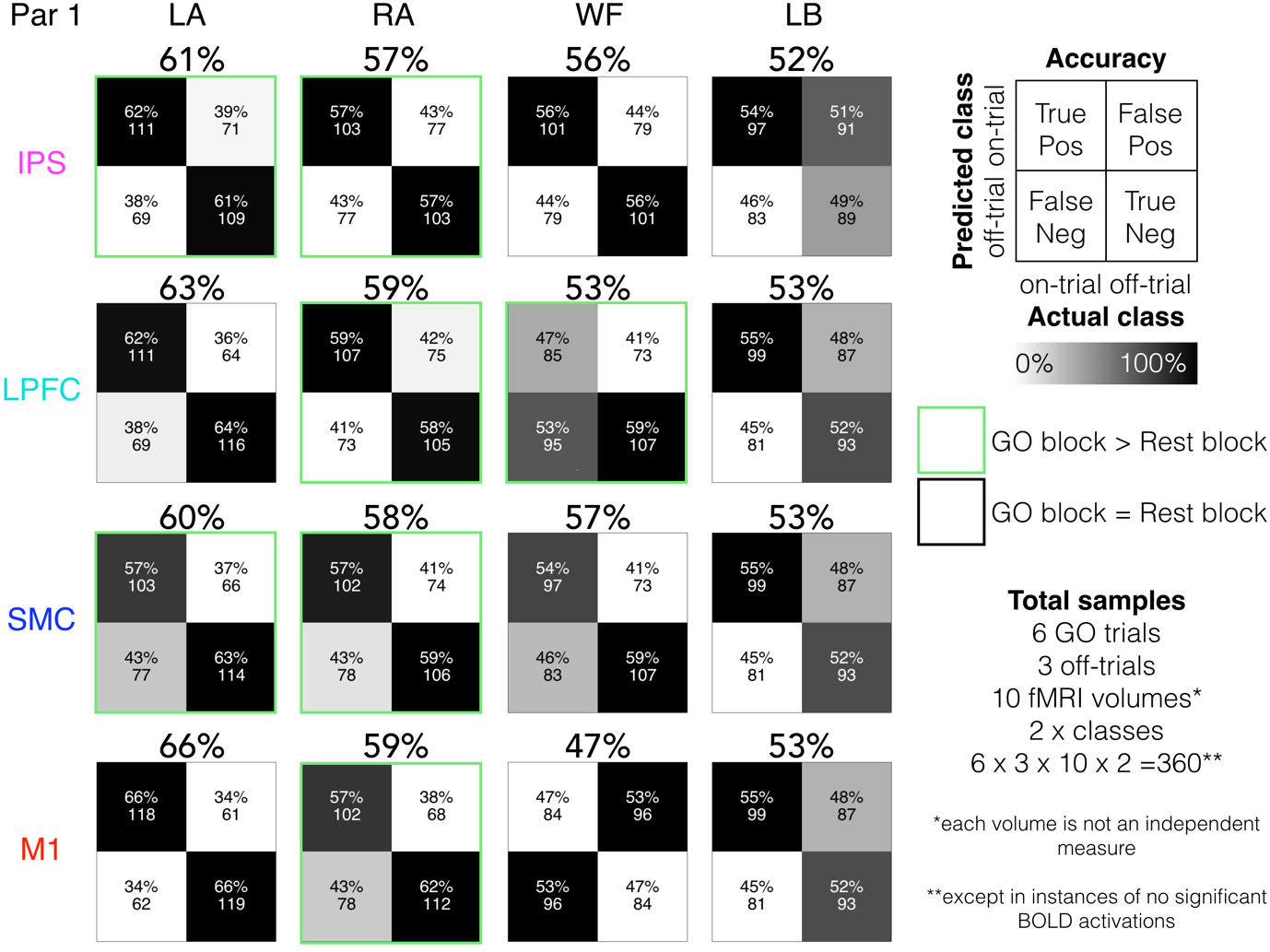

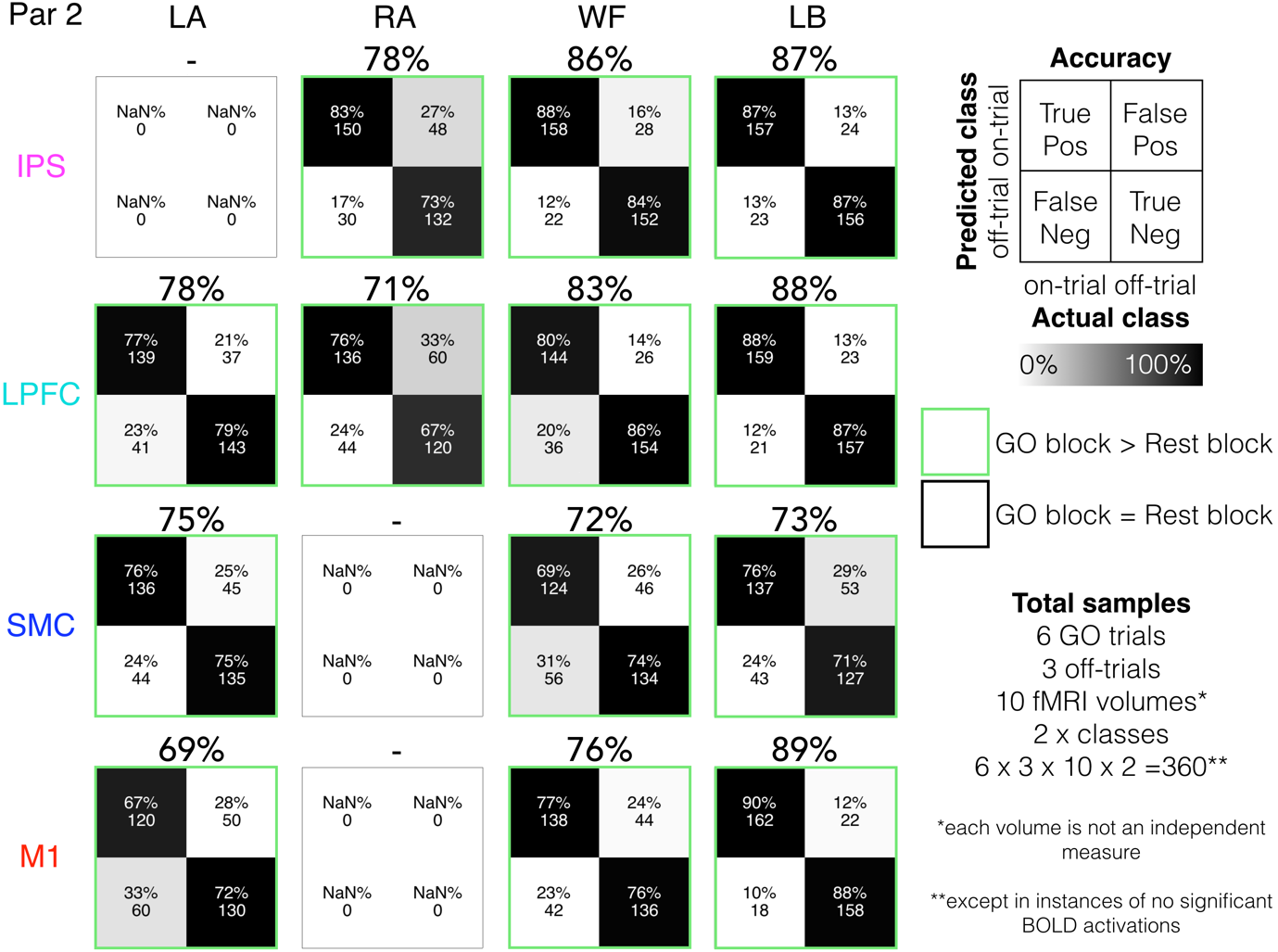


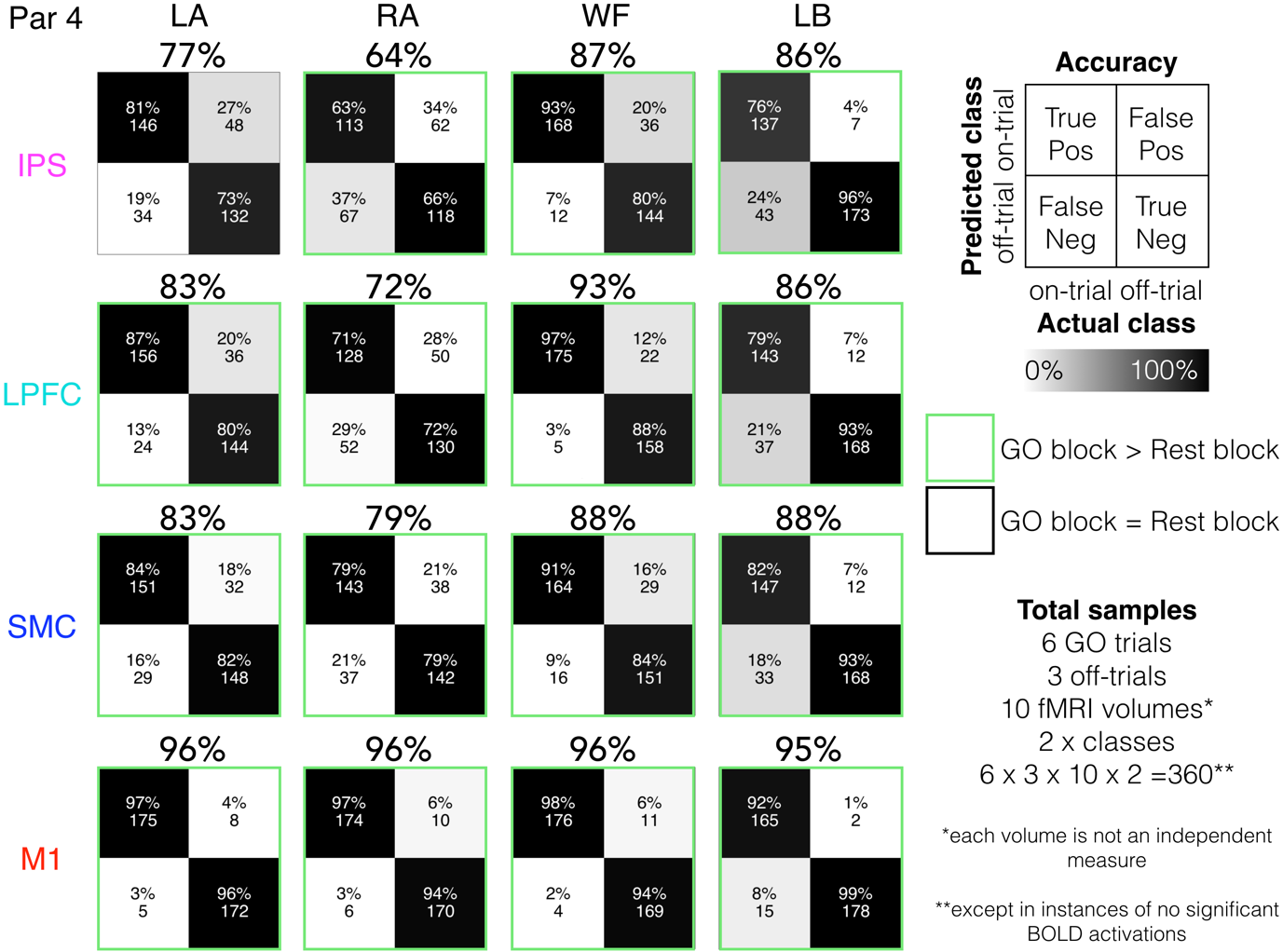

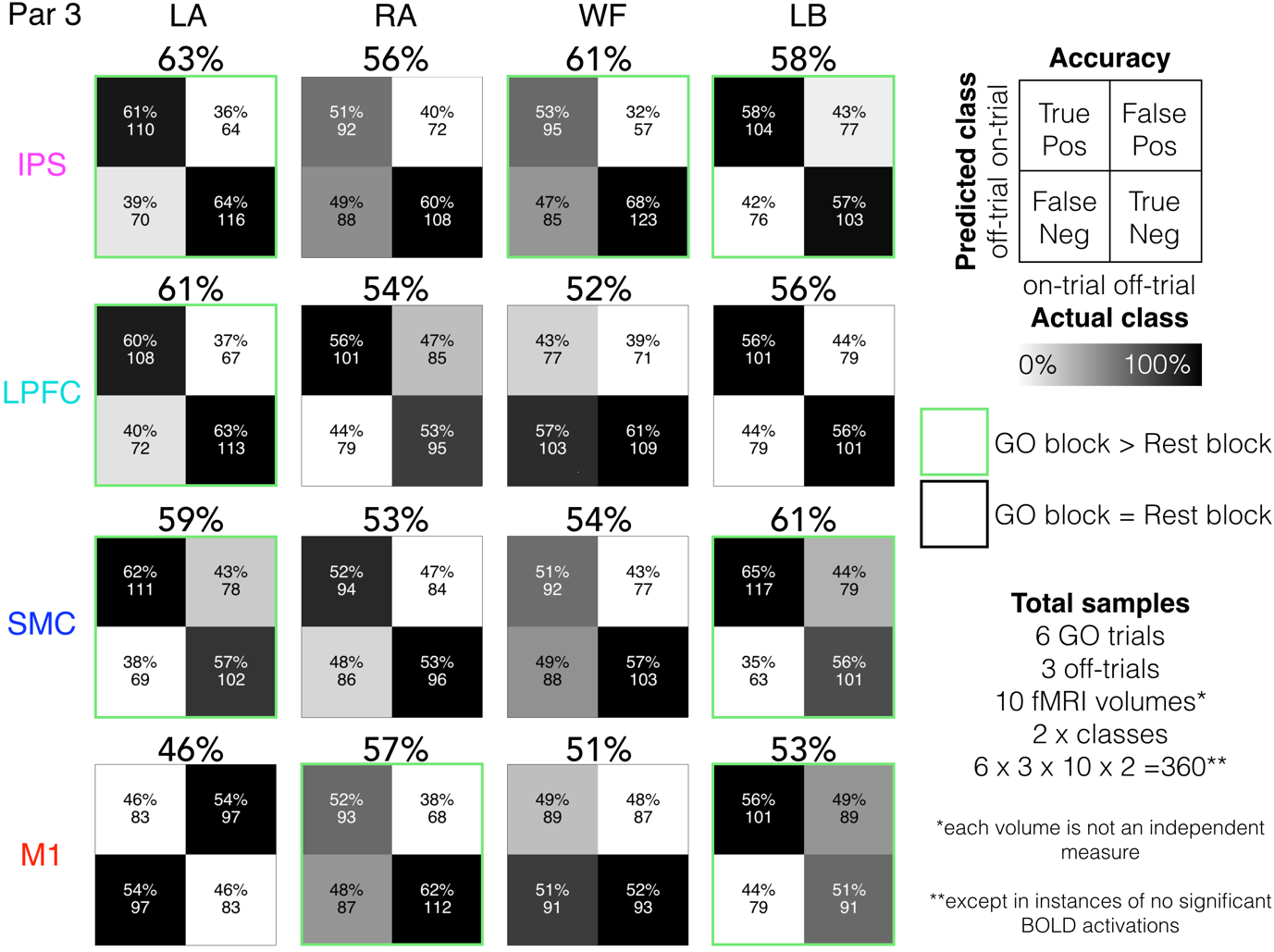


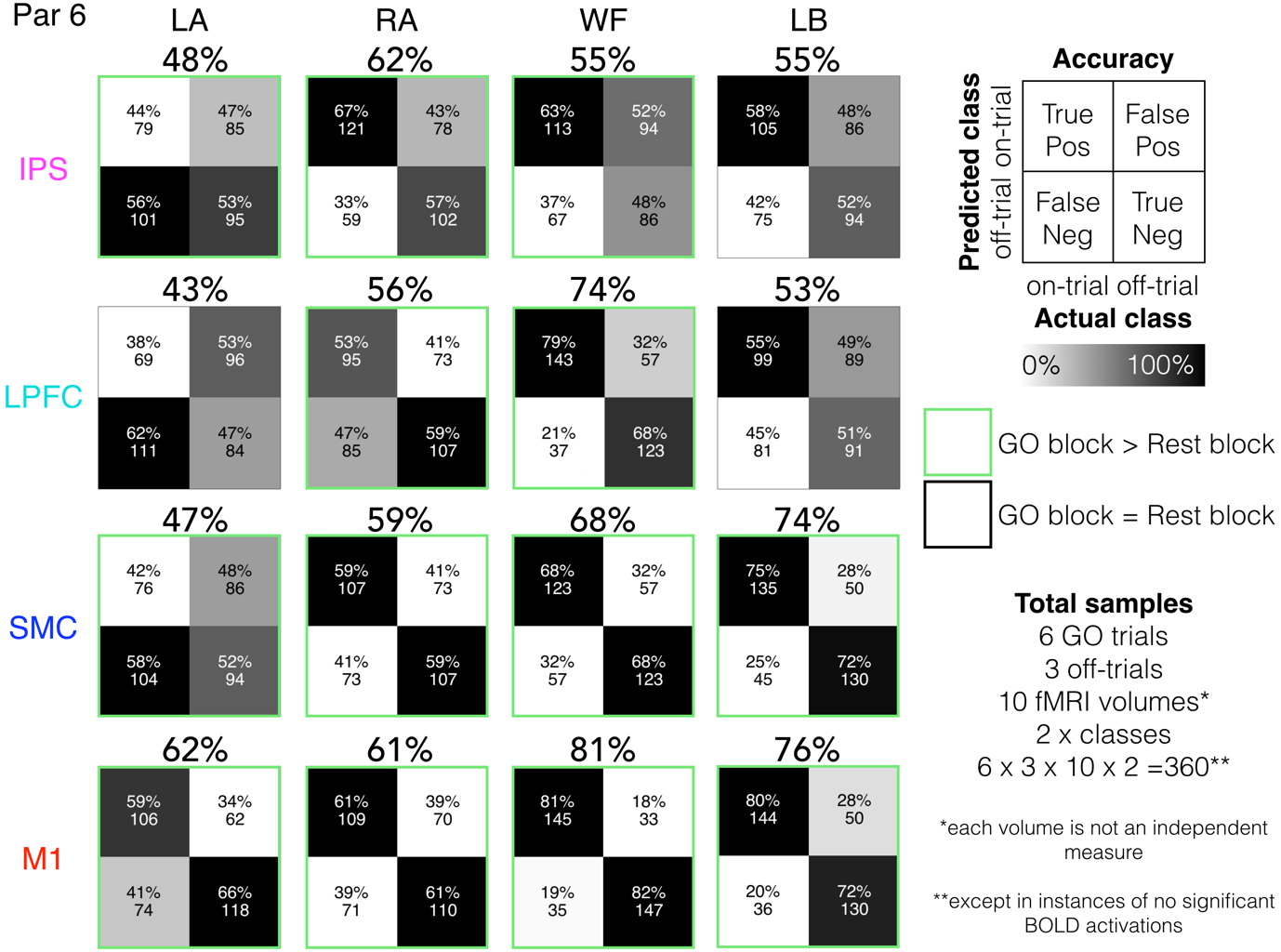

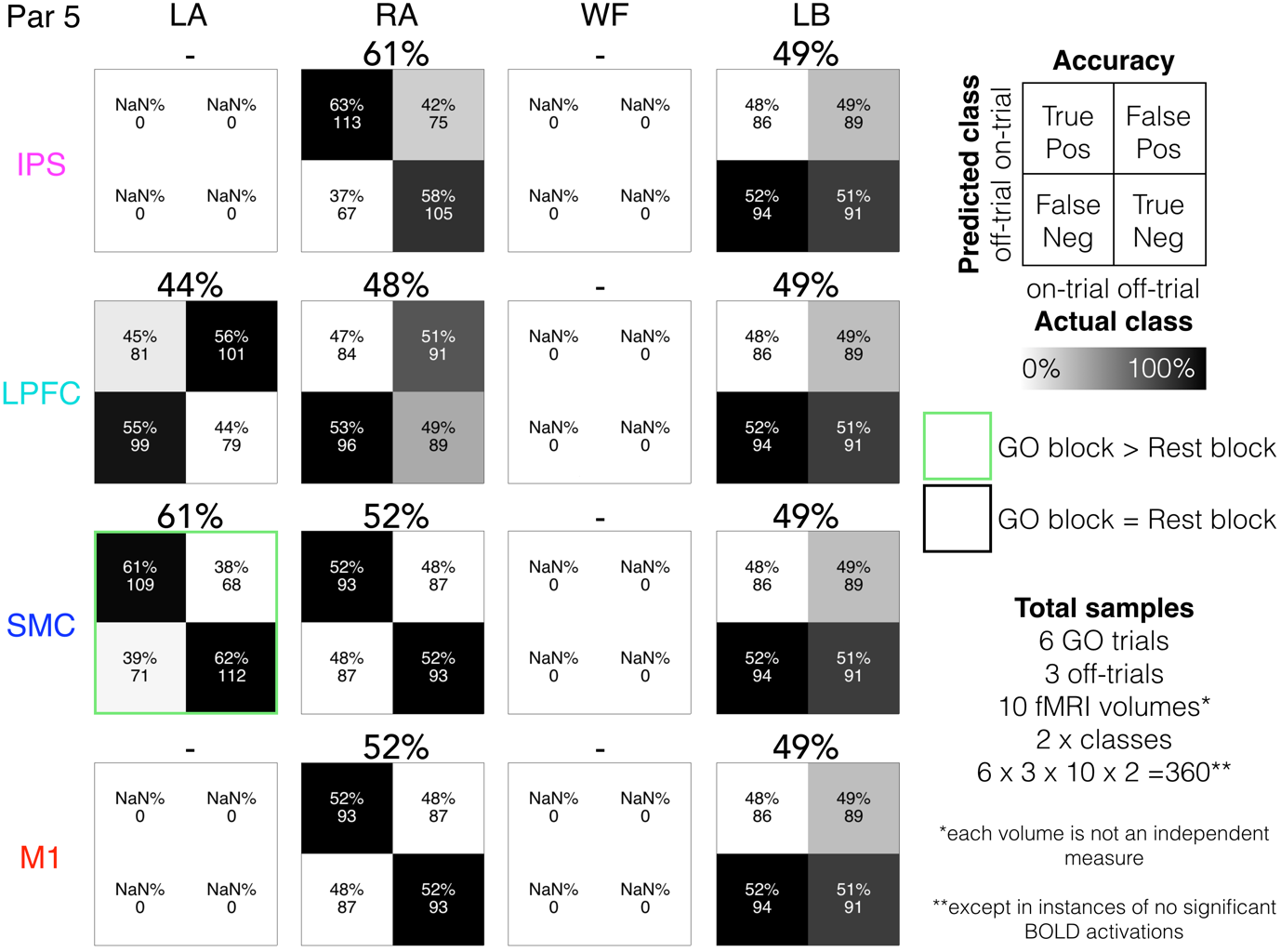


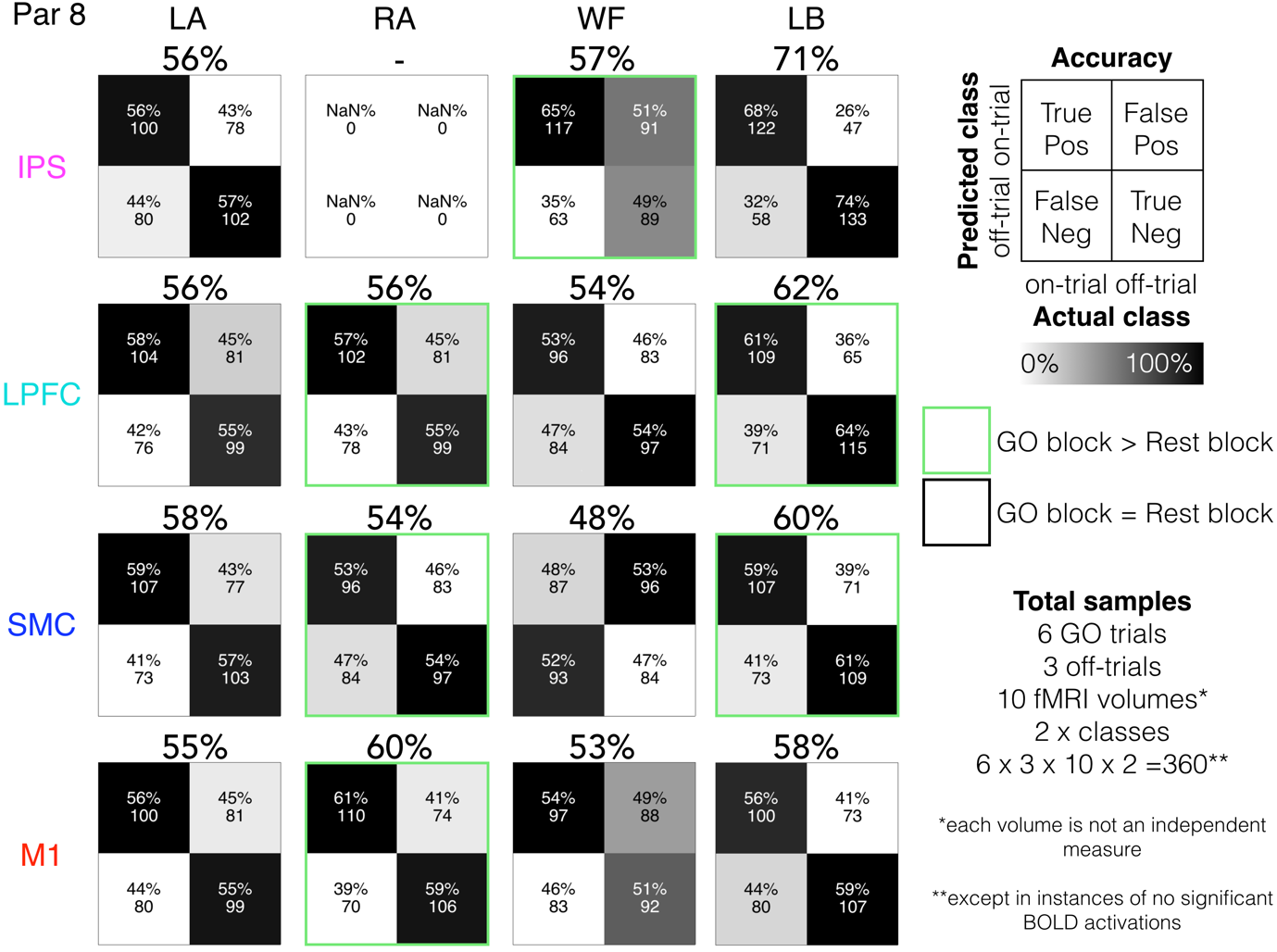

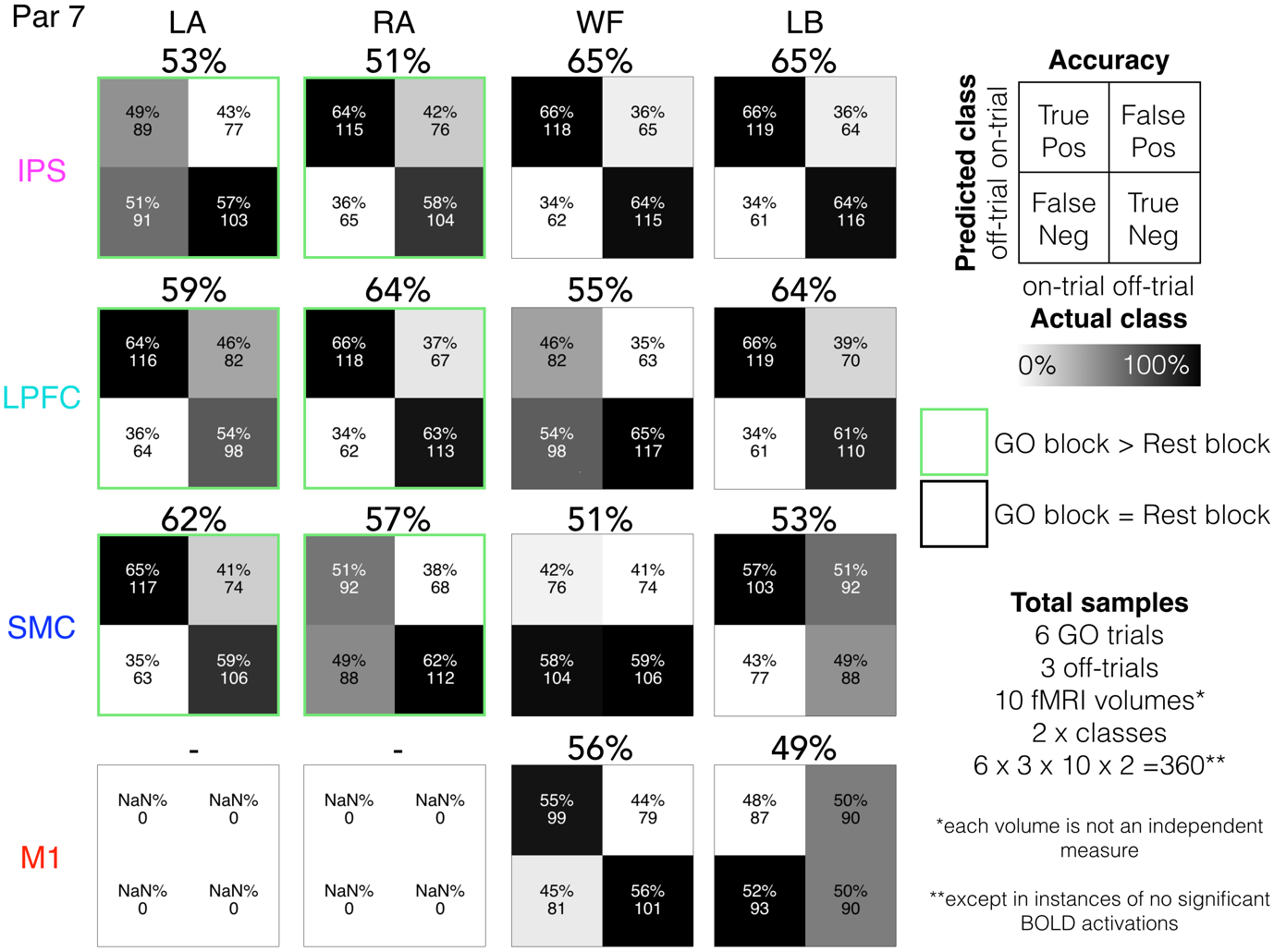


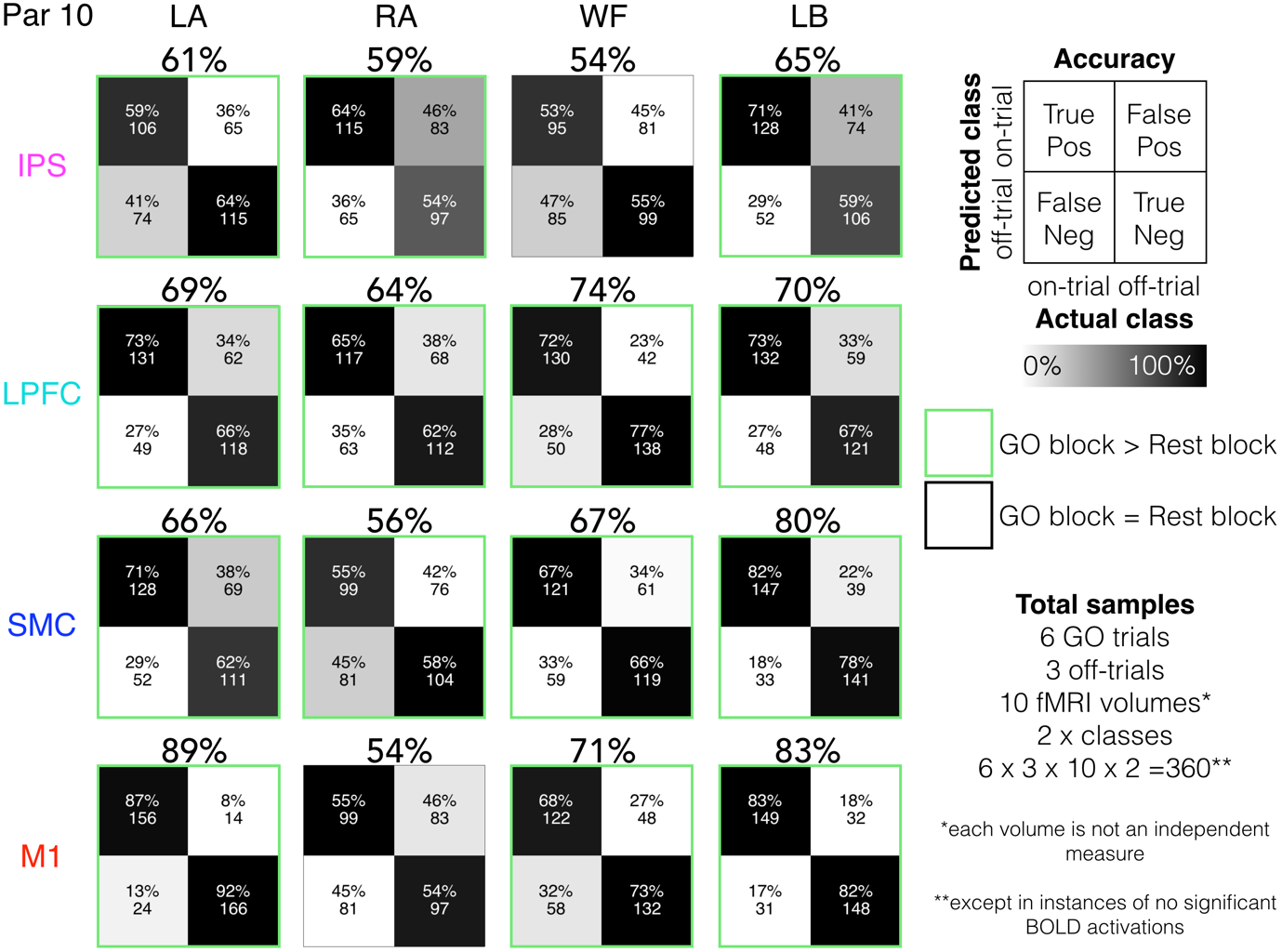

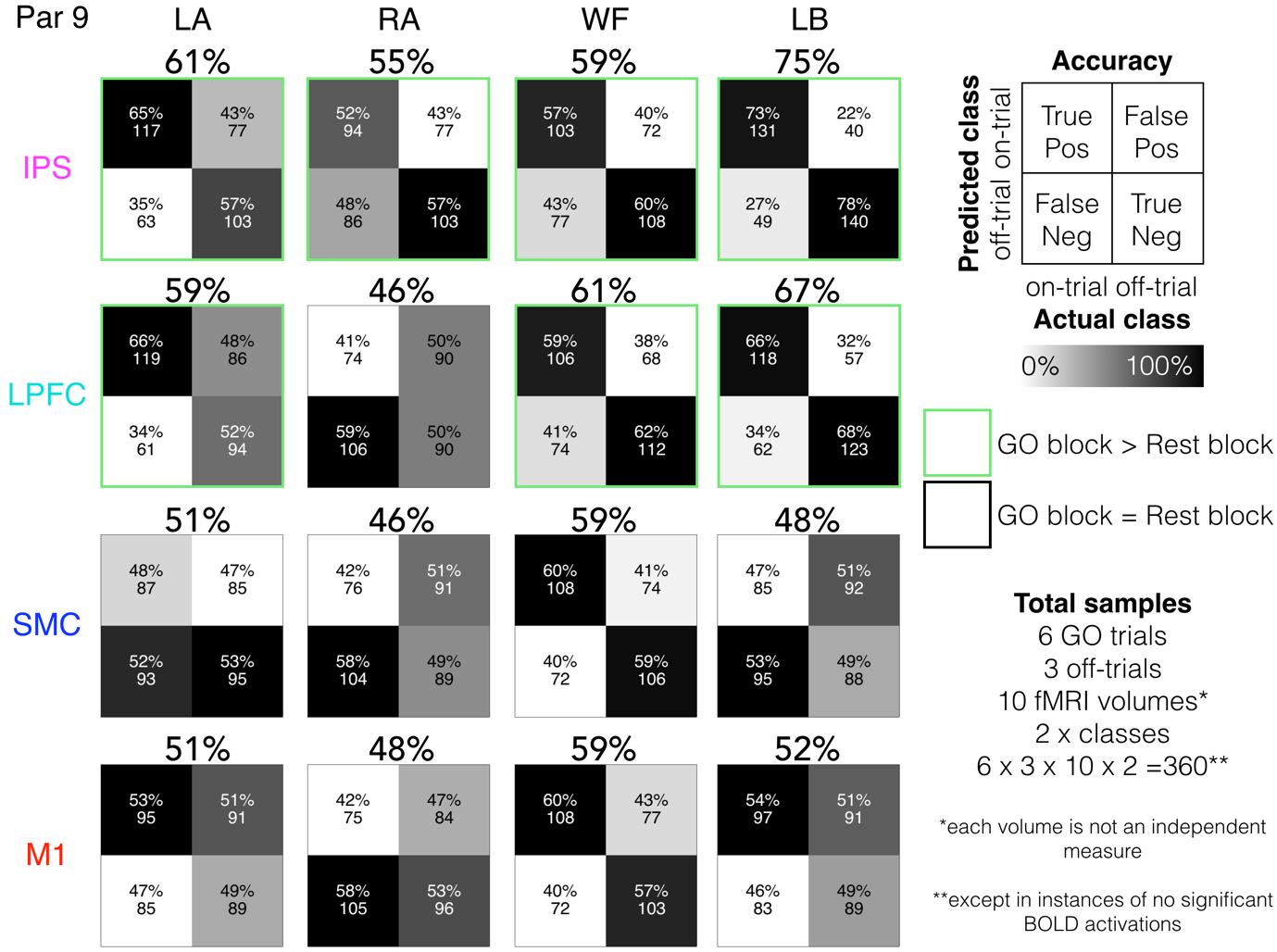


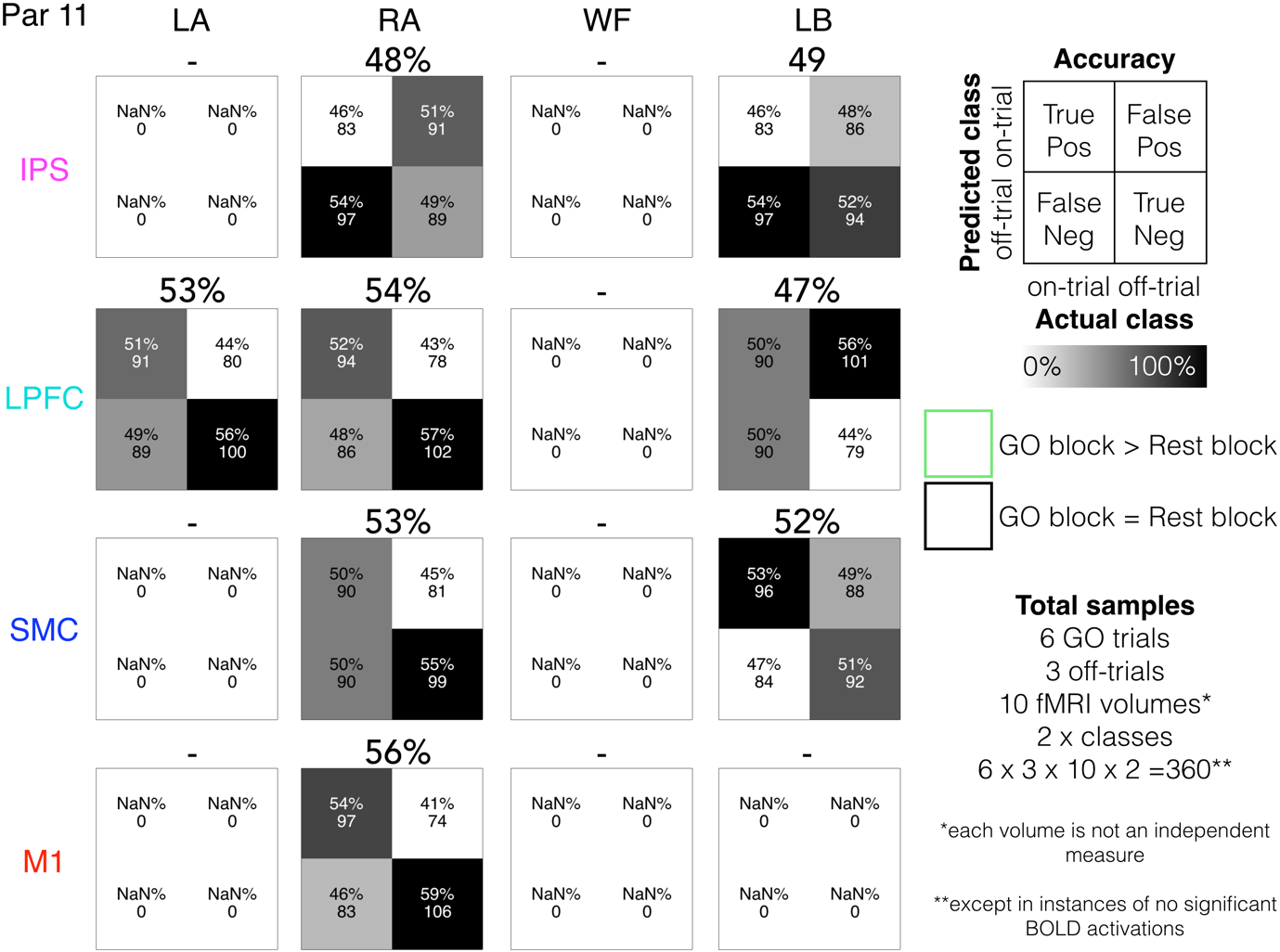


**Supplementary Fig. 1. Confusion matrix and decoding performance of unimodal classification decoding performance for each individual.** Selective imagined movement of the conditions, left ankle, right ankle, walk forward, and lean back, could be decoded against each other at varying levels across different participants. Individual-level confusion matrices and decoding performances (i.e., accuracy) yielded during the GO blocks for each condition and region for all 11 participants. Because there were 6 GO trials, 3 off-trials comparisons per on-trial, 10 fMRI volumes and 2 classes (i.e., on and off-trials), there were a total of 360 samples, except in instances where no significant activations were found. Each fMRI volume time-point is not considered as an independent measure. The green squares denote instances where the decoding performances were significantly greater during the GO block compared to the rest block.

**Supplementary Fig. 2. Bimodal classification decoding performance.** Selective imagined movement of the conditions, left ankle, right ankle, walk forward, and lean back, could be decoded against each other across the dorsal motor network regions at the group-level using a bimodal classification approach. Condition-specific participant average confusion matrices and decoding performances yielded during GO blocks across specific bimodal combination of the dorsal motor network regions. There were total of 3960 samples per comparison given that there were 11 participants, 6 GO trials, 3 off-trials per on-trial, 10 time-points (fMRI volumes) and 2 classes (i.e., on and off-trial) across the GO block. However, each time point is not independent from each other. The green squares denote instances where the decoding performances were significantly greater during the GO block compared to the rest block.


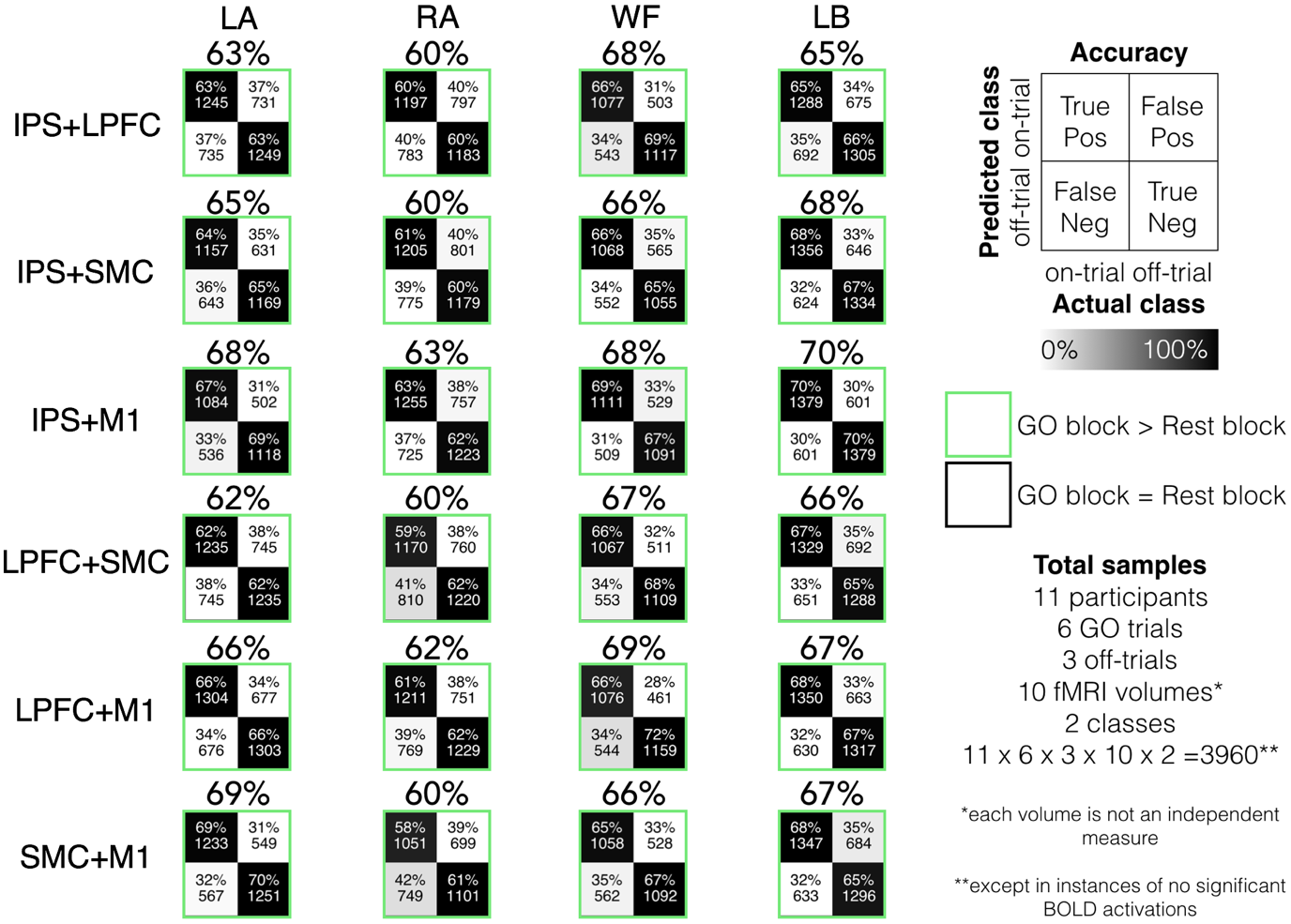

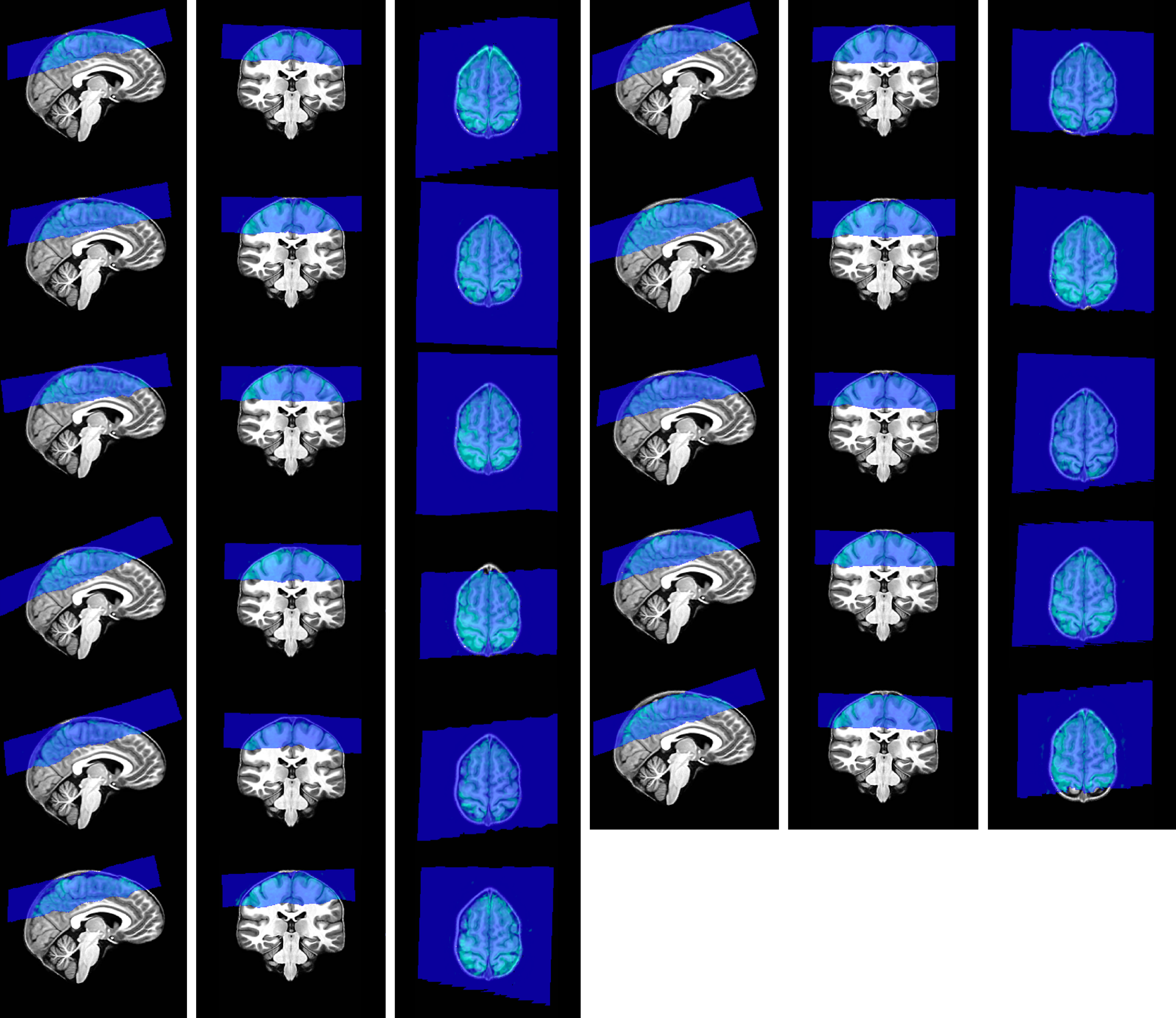


**Par. 01**

**Par. 02**

**Par. 03**

**Par. 04**

**Par. 06**

**Par. 05**

**Supplementary Fig. 3. Partial-coverage functional image of each participant co-registered to the study-specific template space.** Each participant’s partial-coverage functional images (semi-opaque blue) were co-registered to the study-specific template (background image) using OPFAP.


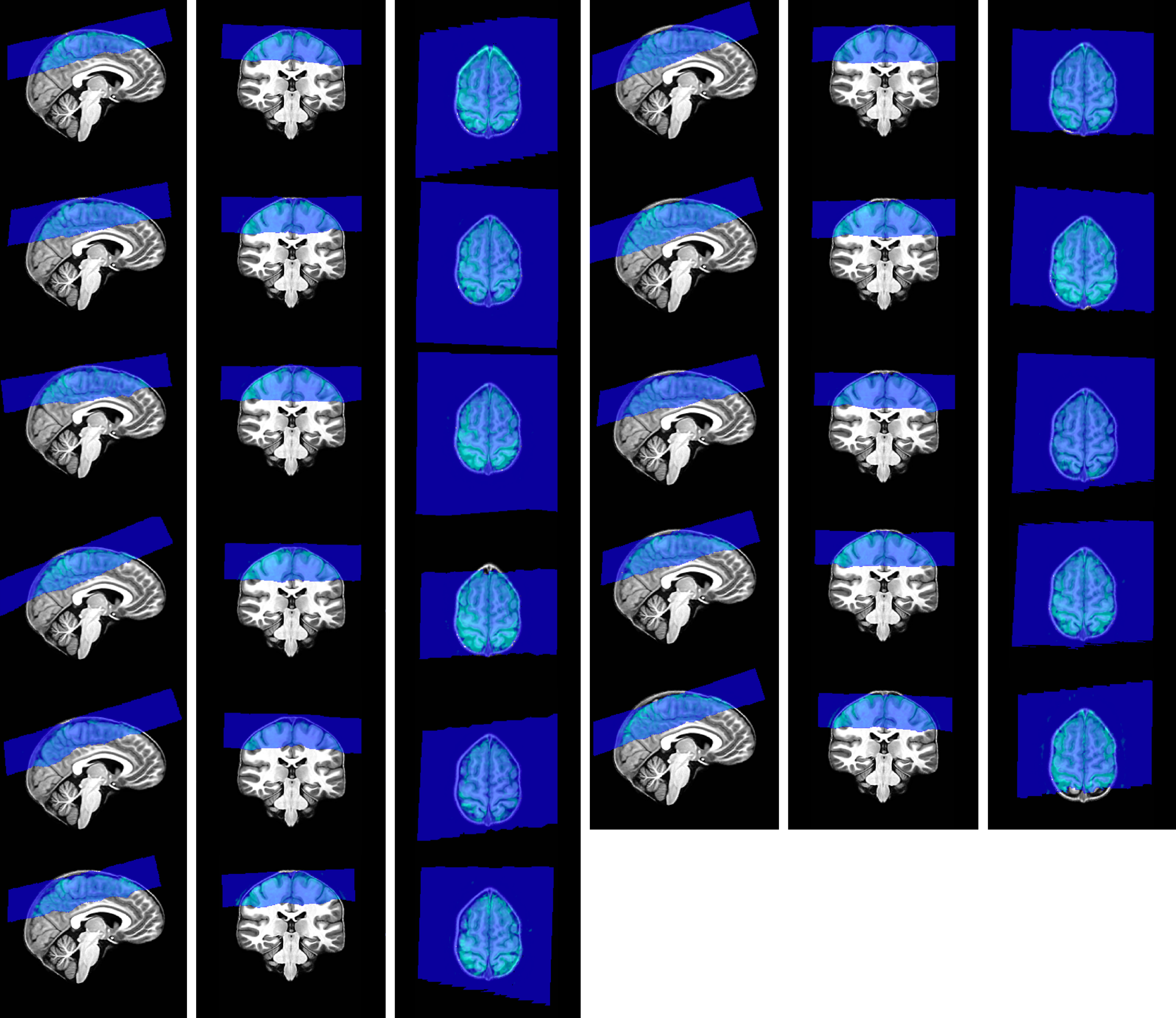


**Par. 07**

**Par. 08**

**Par. 09**

**Par. 11**

**Par. 10**
